# Quantum Sensing of Free Radicals in Primary Human Granulosa Cells with Nanoscale Resolution

**DOI:** 10.1021/acscentsci.3c00747

**Published:** 2023-08-30

**Authors:** Nuan Lin, Koen van Zomeren, Teelkien van Veen, Aldona Mzyk, Yue Zhang, Xiaoling Zhou, Torsten Plosch, Uwe J. F. Tietge, Astrid Cantineau, Annemieke Hoek, Romana Schirhagl

**Affiliations:** † Department of Obstetrics and Gynecology, University of Groningen, University Medical Center Groningen, 9713 GZ Groningen, The Netherlands; ‡ Department of Obstetrics and Gynecology, The First Affiliated Hospital of Shantou University Medical College, 515041 Shantou, China; § Department of Biomedical Engineering, Groningen University, University Medical Center Groningen, Antonius Deusinglaan 1, 9713 AW Groningen, The Netherlands; ∥ Institute of Metallurgy and Materials Science, Polish Academy of Sciences, Reymonta 25, 30-059 Krakow, Poland; ⊥ Center for Reproductive Medicine, Shantou University Medical College, Shantou 515041, China; # Division of Clinical Chemistry, Department of Laboratory Medicine, Karolinska Institute, SE-141 52 Stockholm, Sweden; □ Clinical Chemistry, Karolinska University Laboratory, Karolinska University Hospital, Stockholm, SE-141 86 Stockholm, Sweden

## Abstract

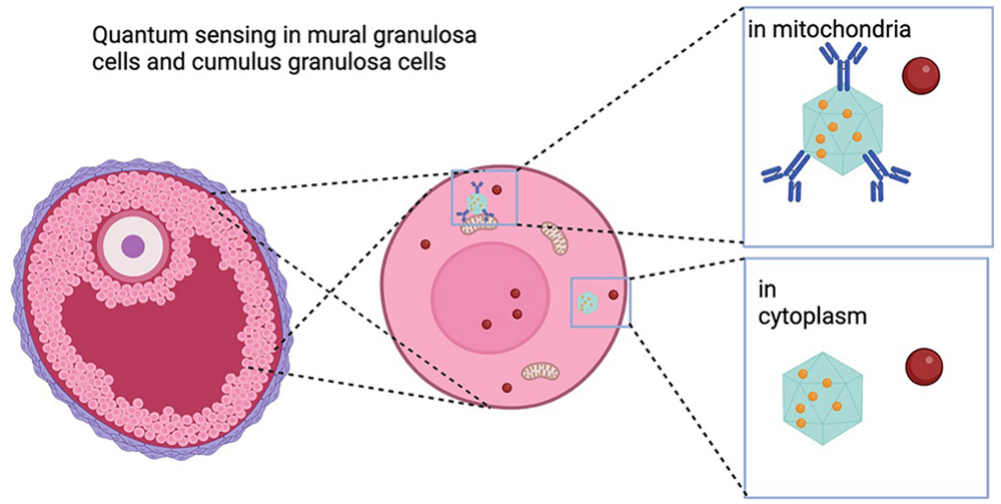

Cumulus granulosa cells (cGCs) and mural granulosa cells (mGCs), although derived from the same precursors, are anatomically and functionally heterogeneous. They are critical for female fertility by supporting oocyte competence and follicular development. There are various techniques used to investigate the role of free radicals in mGCs and cCGs. Yet, temporospatial resolution remains a challenge. We used a quantum sensing approach to study free radical generation at nanoscale in cGCs and mGCs isolated from women undergoing oocyte retrieval during *in vitro* fertilization (IVF). Cells were incubated with bare fluorescent nanodiamonds (FNDs) or mitochondria targeted FNDs to detect free radicals in the cytoplasm and mitochondria. After inducing oxidative stress with menadione, we continued to detect free radical generation for 30 min. We observed an increase in free radical generation in cGCs and mGCs from 10 min on. Although cytoplasmic and mitochondrial free radical levels are indistinguishable in the physiological state in both cGCs and mGCs, the free radical changes measured in mitochondria were significantly larger in both cell types, suggesting mitochondria are sites of free radical generation. Furthermore, we observed later occurrence and a smaller percentage of cytoplasmic free radical change in cGCs, indicating that cGCs may be more resistant to oxidative stress.

## Abstract 2

We used quantum sensing to measure free radical generation in the cytosol and mitochondria of cumulus granulosa cells and mural granulosa cells. We found that these cells react differently when challenged by oxidative stress.

## Introduction

Granulosa cells are somatic cells surrounding and supporting the oocytes in mammalian ovarian follicles. At the antral follicle stage during which a fluid-filled cavity called the antrum is formed, the granulosa cells that originally enclose the oocyte differentiate into 2 distinct subtypes under the control of oocyte-secreted factors: the cumulus granulosa cells (cGCs) that are in intimate metabolic contact with the oocyte via gap junctions and the mural granulosa cells (mGCs) that line the wall of the follicular antrum.^1^ Notably, there is not only anatomical heterogeneity but also functional differences between these two subtypes of cells throughout follicle development. Generally, cGCs play a major role in oocyte growth, development, and meiotic maturation, while mGCs primarily execute an endocrine function and engage in mitosis activity leading to follicular growth.^2^ Distinct gene expression profiles between these two cell types reflect the different physiological functions. For instance, genes encoding steroidogenic enzymes, as well as a range of growth factors and hormone receptors were differentially expressed between mGCs and cGCs in rodents as well as in humans.^3,4^

A physiological level of reactive oxygen species (ROS) plays a key role in the development of oocytes and follicles. In the ovarian follicles ROS are fundamental for oocyte meiotic maturation.^5^ Free radicals, including some of the most reactive ROS molecules, can act as secondary messengers for cellular signaling and are involved in the regulation of ovarian physiological processes, such as ovulation.^6^ A certain amount of oxygen is also required in oocyte meiotic maturation.^7^ However, overabundance of free radicals can lead to oxidative stress, which is detrimental to oocyte meiotic maturation^8^ and associated with reduced female fertility.^9^ Although ROS are inevitable products of aerobic metabolism, lifestyle factors such as obesity and pathological conditions such as endometriosis may also contribute to oxidative stress. The “free radical theory of aging” proposed more than half a century ago has been implicated to be associated with fertility.^10^ Emerging evidence supports the central role of oxidative stress in age-related oocyte quality decline such as disturbed meiotic spindle formation that is responsible for chromosomal segregation leading to a higher chance of aneuploid oocytes.^11^ Conceivably, as cells surrounding and supporting the oocytes, free radical levels of cGCs and mGCs and their responses to oxidative stress may be associated with oocyte quality. However, whether these two subtypes of granulosa cells respond differently to oxidative stress remains unknown: on the one hand, these cells are derived from the same origin, and some certain oxidative stress biomarkers in both kinds of cells have been indicated to strongly associate with oocyte developmental competence and even embryo quality;^12^ on the other hand, the heterogeneity between mCGs and cCGs in many different biological aspects is increasingly recognized.^2,3^

To date, free radical detection in biological samples remains a great challenge in practice due to the short lifespans and low abundance.^13^ Electron spin resonance (ESR), which is considered as the gold standard for direct free radical detection, is still faced with the problem that free radicals in biological samples are naturally at a low steady-state concentration.^14^ Although different indirect techniques have been developed and applied for the measurement of free radical levels in granulosa cells,^15^ a temporospatial measurement with single cell resolution has never been achieved. By measurement of the signal generated from redox interaction or oxidative cell damage instead of the radicals themselves, indirect detection is not able to obtain any spatial information or single-cell resolution. In addition, some indirect approaches are based on a free-radical dye reaction and subsequent fluorescent molecule generation. As a result, these dye-based methods suffer from the risks of photobleaching over time and, thus, are not suitable for real-time and longer measurements. Moreover, they reveal the history of free radical generation in samples rather than the current levels.^16^

We use a method based on negatively charged nitrogen vacancy (NV^–^) defects of fluorescent nanodiamonds (FNDs).^17^ Due to their stable fluorescence, they can be used for long-term tracking and labeling.^18^ NV centers also change their optical properties in response to their magnetic surrounding.^19^ Importantly, these FNDs are also excellently biocompatible.^20^ This method offers a new way for direct free radical measurement in real-time and at a subcellular level. NV center-based sensing has already been used for applications in 2-dimensional materials or magnetic characterization of materials under high hydrostatic pressures, sensing of nanoscale temperature,^21^ magnetic nanostructures,^22^ or paramagnetic ions;^23,24^ NV centers are traditionally utilized in physics while their application in biological fields is less explored. NV centers can “feel” magnetic noise from free radicals and convert it into an optical signal. With this method free radical sensing on a subcellular level has been demonstrated in a variety of mammalian cells^25−27^ as well as yeast or bacteria,^28^ suggesting its potential application in granulosa cells.

In the current study, we aim to investigate if diamond-based relaxometry can be applied to measure free radicals in human mGCs and cGCs at subcellular levels in real-time. Further, we aim to test whether cGCs and mGCs respond differently to induced oxidative stress at a subcellular level.

## Results

**Figure 1:**
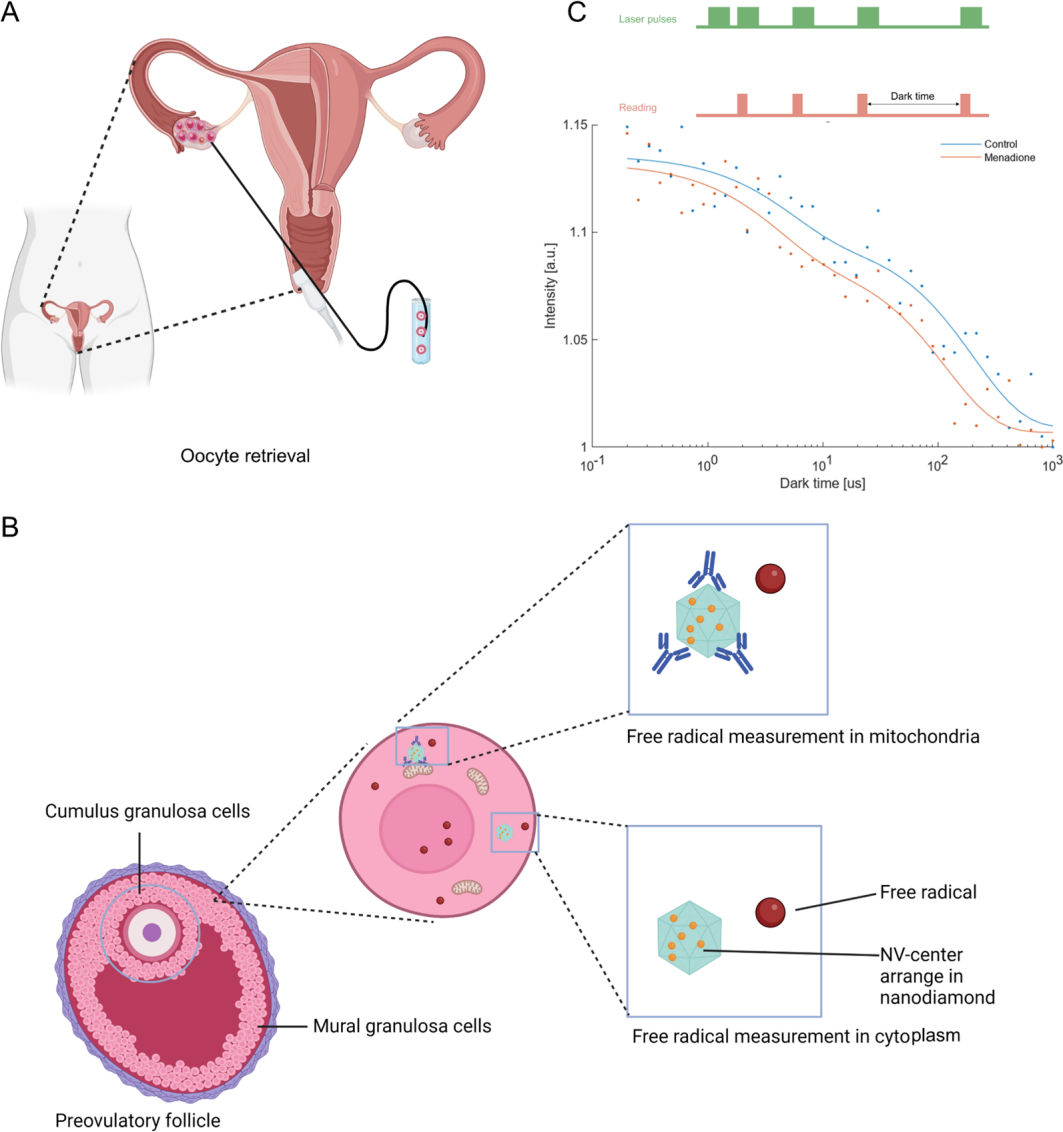
Schematic summary of applying relaxometry to probe free radicals in human granulosa cells by using fluorescence nanodiamond (FND). (A) Oocyte retrieval was performed 36 h after luteinizing hormone (LH) in females who were planned for *in vitro* fertilization (IVF) for fertility problems. Granulosa cells are collected during this procedure. (B) Cumulus granulosa cells (cGCs) and mural granulosa cells (mGCs) were isolated from preovulatory follicles separately, followed by culture and incubation with FNDs. Two subtypes of FNDs were used, bare-FNDs (directed to cytoplasm) and aVDAC2-FNDs (directed to mitochondria) for 24 h before relaxometry; (C) The raw data for representative T1 relaxation curves are shown. These were generated from different dark times plotted against the fluorescence intensity. The inset presents the pulsing sequence used in relaxometry. The green blocks indicate when the laser was on, while the red blocks indicate when the photoluminescence (PL) from the FND was read out.

Figure 1 shows an outline of the quantum sensing experiments that were conducted in this study on cGCs and mGCs isolated from the preovulatory follicles of females during an IVF procedure.

### Characterization of FNDs and Identification of Human Primary Granulosa Cells

To measure free radicals on a subcellular level, two kinds of particles were applied: uncoated FNDs (bare-FNDs) that are expected to be in the cytoplasm at the timing of the measurement, and FNDs coated with physically adsorbed anti-VDAC2 antibodies which bind to voltage-dependent anion channel isoform 2 (aVDAC2-FNDs) that are targeted to the mitochondrial outer membrane as previously described.^26^

The sizes and zeta potentials of bare-FNDs and aVDAC2-FNDs are shown in Figure S1. To exclude a potential contribution of contaminating cells, such as not fully removed red blood cells or immune cells from human follicular fluid samples, identification of human primary granulosa cells was performed by flow cytometry. Follicle stimulating hormone receptor (FSHR) was used as a granulosa cell biomarker due to its high specificity for granulosa cell demonstrated by previous studies.^29^ As flow cytometry plots showed, proportions of FSHR ^+^cGC were as high as 99.46% after isolation (Figure S2). Proportions of FSHR ^+^mGC were 19.19% before Percoll purification and strainer filtering but reached as high as 97.3% afterward (Figure S3), suggesting high purity of both cGCs and mGCs.

### FNDs Do Not Affect Mural and Cumulus Granulosa Cell Viability and Intracellular Reactive Oxygen Species (ROS) levels

**Figure 2:**
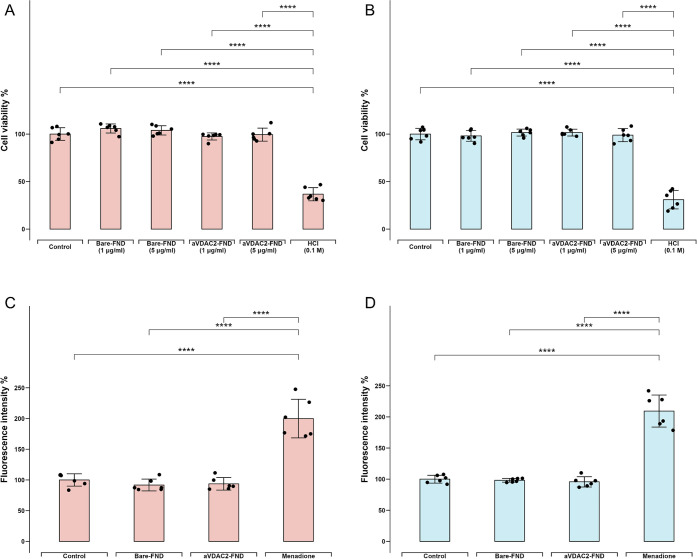
Effects on FNDs on cell viability and intracellular reactive oxygen species (ROS). Cell viabilities were determined by a thiazolyl blue tetrazolium bromide (MTT) assay after incubation with low bare-FND and aVDAC2-FND concentration (1 μg/mL), high bare-FND and aVDAC2-FND concentration (5 μg/mL) and HCl (0.1 M) respectively in cGCs (A) and mGCs (B). DCFHDA assay shows intracellular ROS generation after incubation with bare-FNDs (1 μg/mL), aVDAC2-FNDs (1 μg/mL), and menadione (5 μM) for 24 h in cGCs (C) and mGCs (D). 100% represents a control without any stimuli exposure. The experiment was repeated for cells from six patients, and error bars represent the standard deviations. The data were analyzed by using one-way ANOVA followed by a Tukey post hoc test in comparison to the control groups. ****p < 0.0001.

To confirm that FNDs do not affect metabolic activity and therefore cell viability, an MTT assay (3-[4,5-dimethylthiazol-2-yl]-2,5 diphenyl tetrazolium bromide) was performed. mGCs and cGCs were incubated with different concentrations of bare-FNDs (1 and 5 μg/mL), aVDAC2- FNDs (1 and 5 μg/mL), or HCl for 24 h, respectively. HCl (0.1 M) was used as a positive control, as it induces cell death. There are no differences between untreated cells and the groups exposed to FNDs either at a concentration that we later applied in this study (1 μg/mL) or a concentration that is relatively high for relaxometry (5 μg/mL) (*p* > 0.05, Figure 2A–B), suggesting a good biocompatibility of FNDs in human primary GCs. To test if FNDs induce changes in the intracellular ROS level, a 2′,7′-dichlorodihydrofluorescein diacetate (DCFHDA) assay was performed. Specifically, cGCs and mGCs were incubated with bare FNDs (1 μg/mL), aVADC2 coated FNDs (1 μg/mL) or menadione (10 μM) for 24 h. Menadione was used as a positive control, as it induces intracellular ROS generation. There are no differences between the negative controls and the cells exposed to bare or aVADC2 coated FNDs (*p* > 0.05, Figure 2C–D), indicating that bare and aVADC2 coated FNDs do not affect intracellular ROS levels in cGCs and mGCs and thus can be used for different measurements in these cells.

### Diamond Uptake and Localization in the GCs

Before relaxometry experiments, the uptake of FNDs or aVDAC2-FNDs (1 μg/mL) by GCs after incubation for 2 and 24 h was evaluated with confocal z-scans. Typical images of bare-FNDs and aVDAC2-FNDs uptake by cGCs and mGCs following different incubation times are shown in Figure S4. More aVDAC2-coated FNDs are found inside cells in comparison to bare-FNDs after incubation for 24 h in cGCs (*p* < 0.05) and mGCs (*p* < 0.001) (Figure S5A). Although the uptake of bare-FNDs by cGCs and mGCs was comparable (*p* > 0.05), the number of aVDAC2-FNDs was significantly higher in mGCs compared to that of bare-FNDs (*p* < 0.01) (Figure S5A).

**Figure 3:**
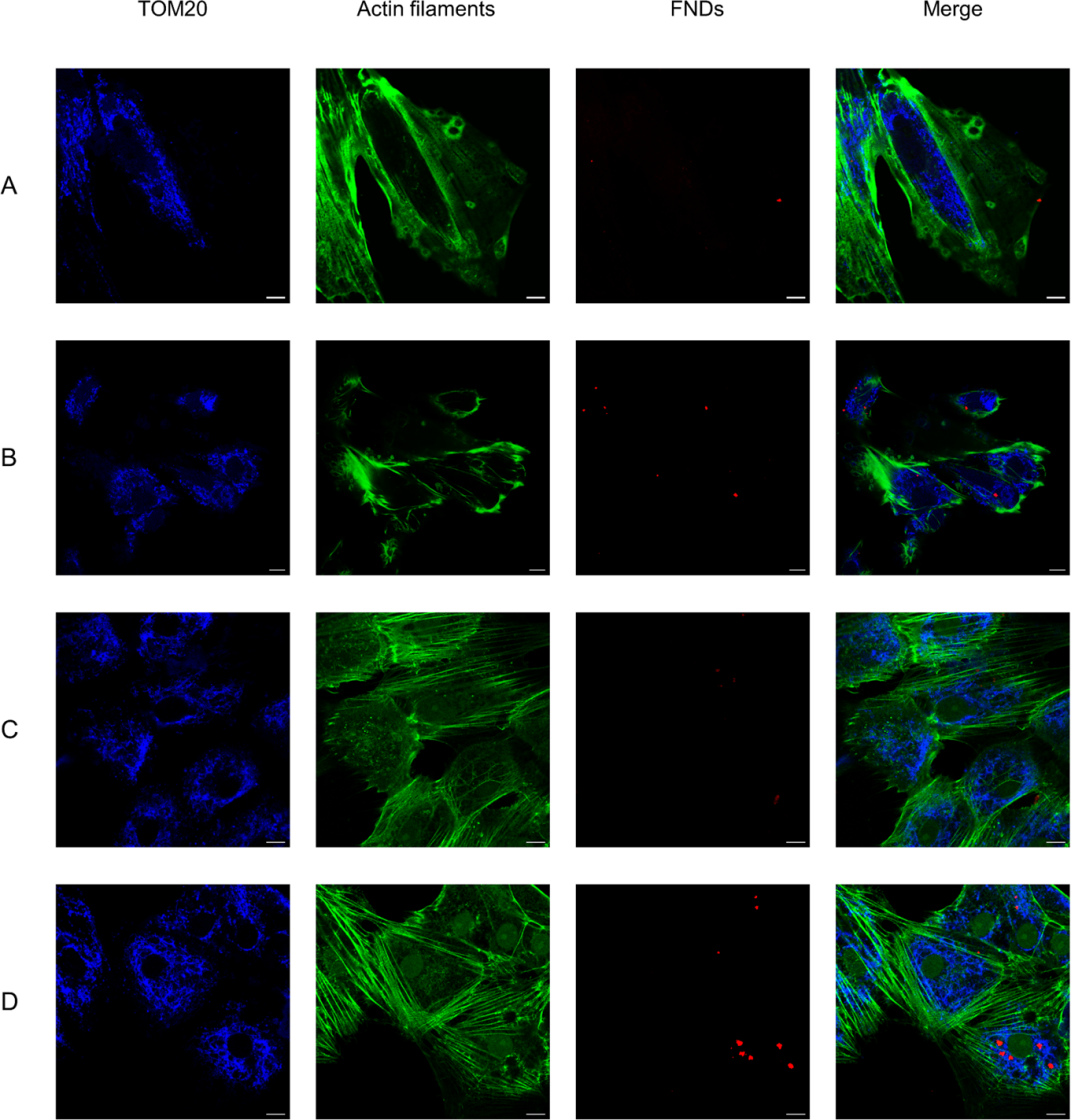
Subcellular location of FNDs revealed by confocal microscopy. Bare-FNDs (1 μg/mL) and aVDAC2-FND (1 μg/mL) were incubated with cGCs (A–B) and mGCs (C–D) for 24 h. Tom20 antibody, an outer mitochondrial membrane biomarker, was used to show mitochondria. Color code: blue, Tom20; green, Phalloidin-FITC, staining actin filaments (also known as F-actin); red, bare-FND or aVDAC2-FND. The scale bar is 10 μm.

To explore the localization of bare FNDs and aVDAC2-FNDs, the colocalization of bare FNDs or aVDAC2-FNDs with *translocase of the outer mitochondrial membrane 20* (Tom20) was observed by confocal microscopy. As shown in Figure 3, bare-FNDs colocalize less with TOM20 but are always in the proximity of actin filaments, whereas aVDAC2-FNDs are prone to colocalize with TOM20, suggesting localization at mitochondria.

### Bare- and aVADC2-FNDs Can Be Applied for Real-Time Cytoplasmic and Mitochondrial Free Radical Detection in cGCs

As shown in the fluorescent images acquired by our homemade relaxometer (Figure S5B), the FND (red arrow) is very bright in comparison to the autofluorescence of the cell.

Before relaxometry measurements, toxicity of menadione at different concentrations was evaluated by MTT assay, and HCl served as a positive control. As shown in Figure S6A–B, incubation with 10 μM of menadione for 30 min was within the safe range for both cGCs and mGCs. To exclude the potential effects of DMSO-dissolved menadione on FNDs, relaxometry measurement of FNDs was performed in the absence of cells. As shown in Figure S6C, no significant T1 change was observed when DMSO-dissolved menadione was added to FNDs in the absence of cells.

**Figure 4:**
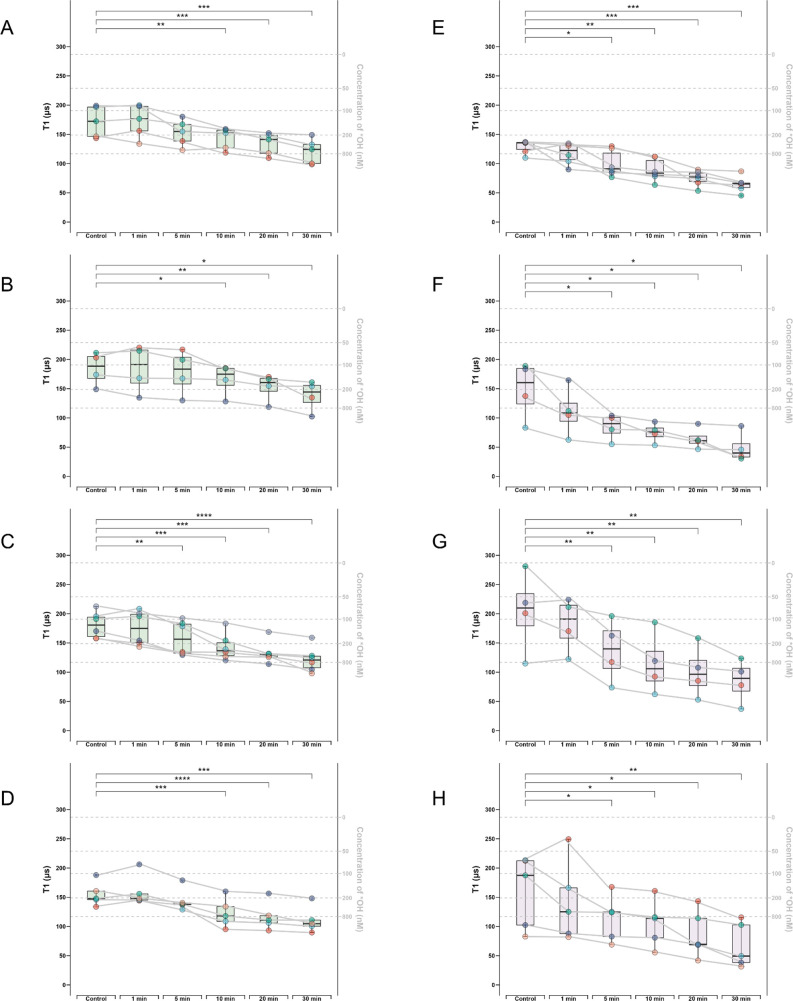
Box-whisker plots shows real-time free radical change determined by T1 after menadione treatment in cGCs from 4 different patients. For each patient, T1 of 4–6 bare-FNDs or aVDAC2-FNDs followed by menadione treatment at different time points (0, 1, 5, 10, 20, 30 min) were measured. Bare-FNDs (left side, boxes of color green) and aVDAC2-FNDs (right side, boxes of color purple) measured in Patient 1 (A–B), Patient 2 (C–D), Patient 3 (E–F), Patient 4 (G–H). The right Y axis represents the estimated radical concentration obtained from previous work.^24^ Each particle is represented by one color, and each curve represents measurements performed on the same particle at different time. The data were analyzed by using a paired *t* test in comparison to the control groups. * *p* < 0.5, ** *p* < 0.01, *** *p* < 0.001, **** *p* < 0.0001.

Both types of nanodiamonds were used in relaxometry measurements to detect real-time free radical level changes after menadione treatment in cGCs from 4 patients (Figure 4). For each type of nanodiamond, 4–6 particles inside the cGCs were selected. For each particle, we tracked the free radical change for 30 min, and time dependent T1 reductions were observed for both FND variants. Among the 4 patients, cGCs from 3 patients present significant changes of T1 values from 10 min on (Figure 4A,B,D), and an earlier significant change of T1 (from 5 min on) is observed in 1 patient (Figure 4C) using bare-FNDs. A decrease in the T1 value corresponds to an increase in the free radical concentration near the nanodiamond sensor. To estimate the radical concentrations equivalent with T1 values, a calibration of *OH radical measurement with known concentrations was obtained from previous work.^24^

To compare T1 measurements with traditional methods, intracellular ROS and mitochondrial superoxide detection assays were performed. The classical intracellular ROS probe, DCFH-DA, was subsequently applied to validate the oxidative stress induced by menadione. A significant intracellular ROS change was observed from 20 min on (*p* < 0.0001, Figure S7A). For the results obtained using aVADC2-FNDs, cGCs from all the patients exhibited a significant T1 decrease from 5 min, and the reduction is time-dependent. A commercial assay kit, MitoSox, was subsequently applied to quantify the mitochondrial superoxide induced by menadione. As shown in Figure S7B, a significant change in mitochondrial superoxide production was observed after incubation with menadione for 20 min (*p* < 0.0001).

### Bare- and aVADC2-FNDs Can Be Applied for Real-Time Cytoplasmic and Mitochondrial Free Radical Detection in mGCs

**Figure 5:**
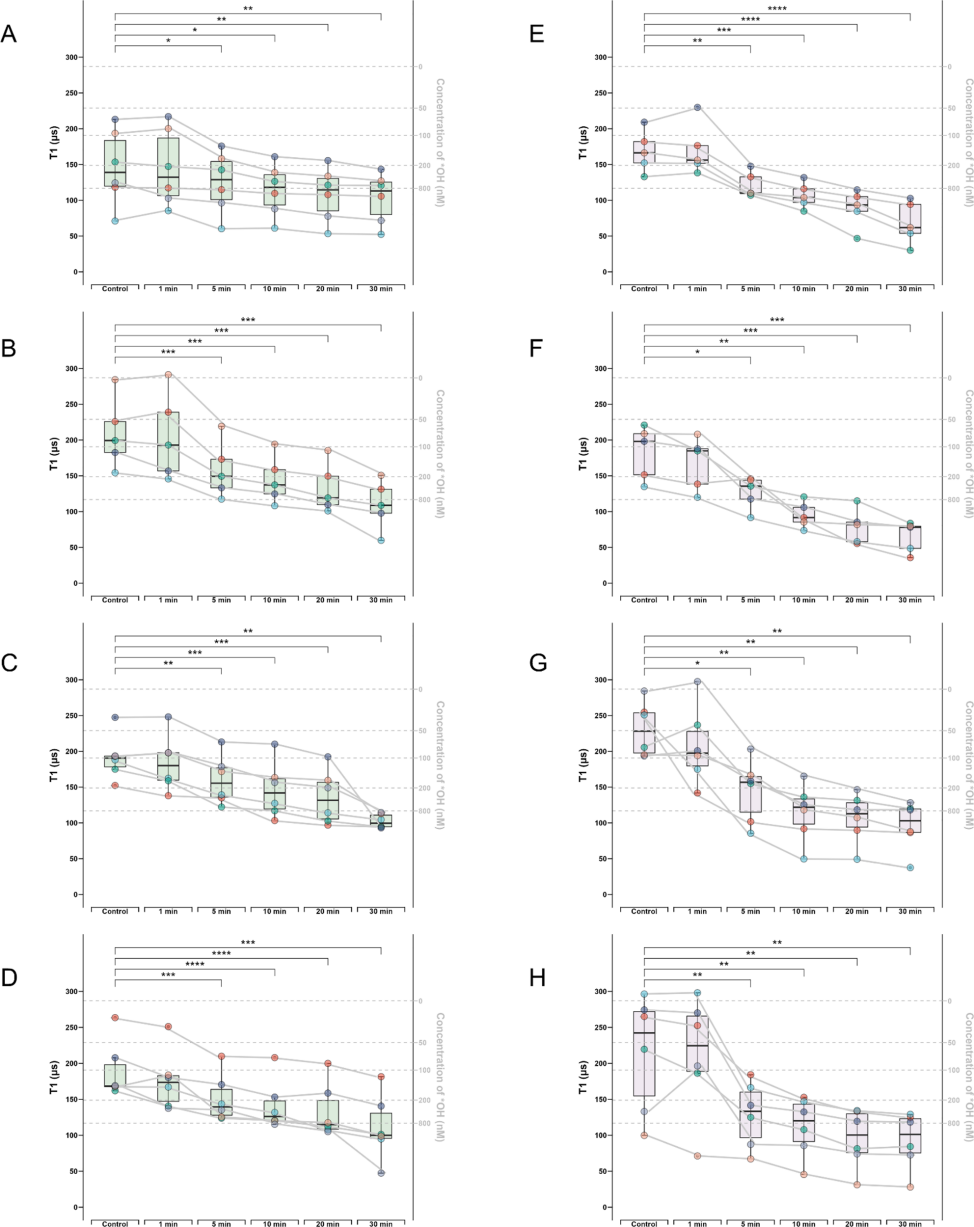
Box-whisker plots shows real-time free radical change determined by T1 after menadione treatment in mGCs from 4 different patients. For each patient, T1 of 4–6 bare-FNDs or aVDAC2-FNDs followed by menadione treatment at different time points (0, 1, 5, 10, 20, 30 min) were measured. Bare-FNDs (left side, boxes of color green) and aVDAC2-FNDs (right side, boxes of color purple) measured in Patient 1 (A–B), Patient 2 (C–D), Patient 3 (E–F), Patient 4 (G–H). The right Y axis represents the estimated radical concentration obtained from previous work.^24^ Each particle is represented by one color, and each curve represents measurements performed on the same particle at different times. The data were analyzed by using a paired *t* test in comparison to the control groups. * *p* < 0.5, ** *p* < 0.01, *** *p* < 0.001, **** *p* < 0.0001.

Similarly, both types of nanodiamonds were used in relaxometry measurements to detect real-time free radical level changes after menadione treatment in mGCs from 4 patients (Figure 5). For each type of nanodiamond, 4–6 particles inside the mGCs were selected according to the criteria mentioned in Methods. For each particle, we tracked the free radical change for 30 min, and time dependent T1 reductions were observed for both FND variants. In mGCs from all patients we observed significant changes of T1 values from 5 min on either using bare-FNDs or aVADC2-FNDs. Consistent with cGC, the reduction of T1 is time dependent. The classical intracellular ROS probe, DCFH-DA, was applied to validate the oxidative stress induced by menadione. Significant changes of intracellular ROS levels were observed from 10 min on (*p* < 0.05, Figure S7C). MitoSox was applied to quantify mitochondrial superoxide induced by menadione. As shown in Figure S7D, significant changes in mitochondrial superoxide production were observed after incubation with menadione for 20 min (*p* < 0.0001).

### Free Radical Changes upon Oxidative Stress Are Different on a Subcellular Level

**Figure 6:**
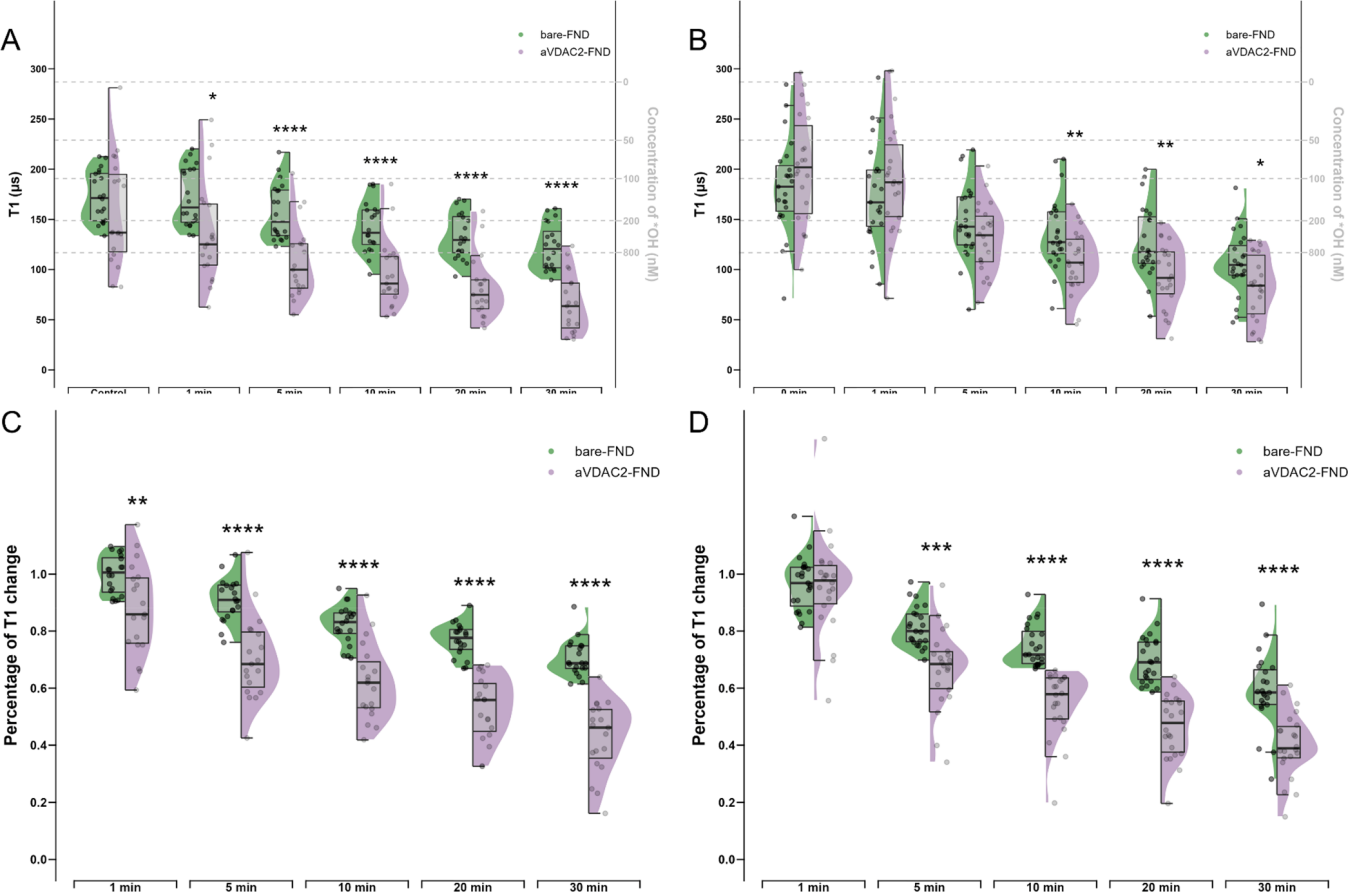
Half-violin plots showing differences between cytoplasm and mitochondrial free radical change in response to menadione at different time points. T1 measurements from all the bare-FNDs and aVDAC2-FNDs were compared to show the differences between cytoplasm and mitochondrial absolute free radical levels at 0, 1, 5, 10, 20, 30 min after menadione treatment in cGCs (A) and mGCs (B). Percentages of T1 changes from all the bare-FNDs and aVDAC2-FNDs were compared to show the differences between cytoplasm and mitochondrial free radical changes at 1, 5, 10, 20, 30 min when compared to the control in cGCs (C) and mGCs (D). Significance was tested by using a *t* test. * *p* < 0.5, ** *p* < 0.01, *** *p* < 0.001, **** *p* < 0.0001.

To investigate if menadione induces free radical changes in the cytoplasm and the mitochondria, we compared the T1 values measured by bare-FNDs and aVADC2-FNDs in the GCs. In both cGCs and mGCs, there were no significant T1 differences between the measurement of bare-FNDs and aVADC2-FNDs in the absence of menadione (*p* > 0.05 at 0 min) (Figure 6A–B). This suggests that cytoplasmic and mitochondrial free radical levels are indistinguishable in the physiological state. However, in the presence of menadione, T1 values measured by aVADC2-FNDs were significantly lower than those measured by bare-FNDs from 1 min on in cGCs (*p* < 0.05 at 1 min and *p* < 0.0001 at 5, 10, 20, 30 min) (Figure 6A) and from 10 min on in mGCs (*p* < 0.01 at 10, 20 min and *p* < 0.05 at 30 min) (Figure 6B). The percentage of T1 reduction was significantly bigger from 5 min on when the FNDs were targeted to mitochondria in both cGC (*p* < 0.0001) (Figure 6C) and mGC (*p* < 0.001) (Figure 6D). These results indicate that mitochondria are the main sites of menadione-induced free radical generation. In addition, in cGCs, the long shape of the purple halves of the violin graph indicated a bigger variance in the mitochondria than that in the cytoplasm, the free radical of which were presented as the green half-violin graph. In general, both bare-FNDs and aVDAC2-FNDs showed bigger T1 variations at physiological states which decreased in time after exposure to menadione. This suggests a nonlinear change in T1 upon oxidative stress. A smaller variation of the T1 values beyond a certain level of oxidative stress may be a result of saturation.

### Free Radical Changes upon Oxidative Stress Differ between Cumulus and Mural Granulosa Cells

**Figure 7:**
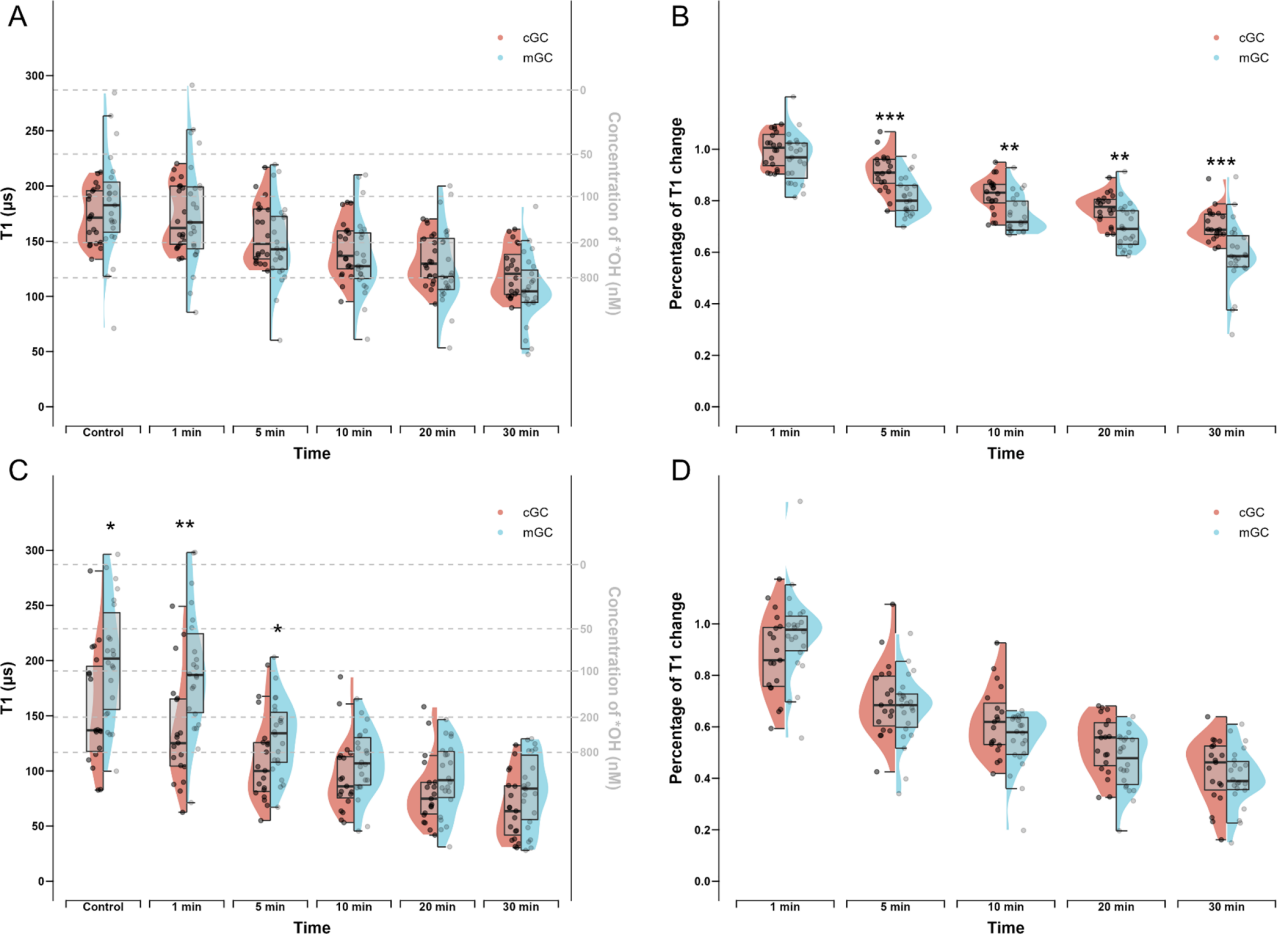
Half-violin plots showing differences between cGCs and mGC free radical levels and changes in response to menadione at different time points. (A) T1 values measured by all the bare-FNDs in cGCs and mGCs were compared to show the differences between cGC and mGC absolute cytoplasm free radical levels at 0, 1, 5, 10, 20, 30 min after menadione treatment. (B) Percentages of T1 changes measured by all the bare-FNDs in cGCs and mGCs were compared to show the differences between cGC and mGC cytoplasm free radical changes at 1, 5, 10, 20, 30 min when compared to the control. (C) T1 measured by all the aVDAC2-FNDs in cGCs and mGCs were compared to show the differences between cGC and mGC absolute mitochondrial free radical levels at 0, 1, 5, 10, 20, 30 min after menadione treatment. (D) Percentages of T1 changes measured by all the aVDAC2-FNDs in cGCs and mGCs were compared to show the differences between cGC and mGC mitochondrial free radical changes at 1, 5, 10, 20, 30 min when compared to the control. Significance was tested by using a *t* test. * *p* < 0.5, ** *p* < 0.01, *** *p* < 0.001, **** *p* < 0.0001.

To investigate if menadione induces free radical changes in cumulus and mural granulosa cells, we compared the absolute T1 values at different time points as well as the T1 change of these two subtypes of cells after exposure to menadione. Using bare-FNDs which measure the cytoplasm free radical levels, we found no significant differences between cGCs and mGCs at any time point (*p* > 0.05) (Figure 7A). However, from 5 min on, the percentage of T1 reduction in mGCs was significantly bigger compared to that in cGCs (*p* < 0.01) (Figure 7B). These results suggest that although the cytoplasmic free radical levels are comparable between cGCs and mGCs either in the basal state or after exposure to oxidative stress, the cGCs are more resistant to oxidative stress. When aVADC2-FNDs were used to measure the mitochondrial free radicals, the absolute T1 values were significantly higher in mGCs at the basal state (*p* < 0.05) and during the early period (at 1 and 5 min) of oxidant exposure (*p* < 0.01 and *p* < 0.05, respectively) (Figure 7C). However, at later time points (at 10, 20, and 30 min), the absolute T1 values between the two types of cells become comparable (*p* > 0.05) (Figure 7C), and no significant percentage of T1 reduction at any time point between cGC and mGCs was observed (*p* > 0.05) (Figure 7D). These results implicate that although the mitochondrial free radical levels are lower in mGC physiologically, upon oxidative stress at first, the mitochondrial free radical response to the oxidant between these two types of cells was similar.

### Influence of the Surrounding Environment on T1 Relaxation

To exclude the possibility that T1 values can be affected by the surrounding environment, such as temperature or stress induced by the measurement conditions themselves during the 30 min measurement, T1 were measured in both kinds of cells using bare-FNDs and aVDAC2-FNDs without menadione. As shown in Figure S8, no significant T1 changes were observed in both cGC and mGCs, either incubated with bare-FNDs or aVDAC2-FNDs for 30 min.

## Discussion

In this study, we aimed to use a quantum sensing approach to probe free radical generation in human cGCs and mGCs. To achieve this goal, we first confirmed that the diamond relaxometry allows for temporospatial free radical measurement in both types of granulosa cells with high biocompatibility and sensitivity. Further, similarities as well as differential free radical responses on subcellular levels were revealed: mitochondria may serve as the main sites of menadione induced free radical generation in both kinds of cells; cGCs may be more resistant to oxidative stress compared to mGCs. Several previous studies have demonstrated that menadione induces intracellular ROS, especially superoxide via one-electron transfer reactions at multiple cellular sites.^30,31^ In this study, menadione is used as an external oxidant to trigger the generation of free radicals in both mGCs and cGCs. The temporospatial property of relaxometry provides us with some interesting biological findings based on the differential ROS generation between cytoplasm and mitochondria as well as cGCs and mGCs upon oxidative stress.

Several cellular organelles, including mitochondria, lysosome, endoplasmic reticulum, are responsible for ROS generation.^32^ Among these, mitochondria are energy powerhouses in most mammalian cells and are considered as the major source of ROS, which results from electron escape from the internal mitochondrial membrane as a natural byproduct of mitochondrial oxidative phosphorylation (OXPHOS) during ATP generation.^33^ In addition, the role of mitochondria in granulosa cell functions has been highlighted in the literature. For instance, mitochondria ATP is the primary source of energy for the FSH-dependent proliferation and differentiation of mouse granulosa cells during folliculogenesis.^34^ Impaired mitochondrial OXPHOS function in granulosa cells was also associated with maternal aging and oocyte incompetence.^35^ Thus, in our model of ROS challenge, mitochondrial ROS changes were compared with cytoplasmic ROS after menadione induction. Interestingly, we found that although cytoplasm and mitochondrial free radical levels are indistinguishable in the physiological state in both kinds of cells, significantly bigger percent changes were observed in mitochondria compared to cytoplasm after 5 min of oxidant induction. This finding suggests that mitochondria may be the major sites of menadione-induced free radical generation, which is in accordance with previous work showing that menadione causes rapid superoxide accumulation in neuronal cells. The authors speculated that this accumulation occurred preferentially in mitochondria of hippocampal neuronal cells.^30^

As described earlier, cGCs are in direct contact with and metabolically coupled with the oocyte through gap junctions. Thus, cGCs have long been believed to be the gatekeepers for the oocyte from its surroundings and play a variety of essential roles in the growth and meiotic maturation of oocytes. It is also known that the oocyte relies on the surrounding cGCs for providing protection against excessive ROS since it does not have the capacity on its own to mobilize all the necessary antioxidant defense mechanisms.^36^ On the other hand, mGCs execute more endocrine rather than defense functions.^2^ Thus, it is not surprising that cGCs are more resistant to oxidative stress after exposure to oxidative stress than mGCs.

There are also some results different from those from previous studies of diamond relaxometry in other cell types. In contrast to previous studies showing higher T1 variability in the macrophage cytoplasm,^26^ we found a bigger variance in the mitochondria than that in the cytoplasm in cGCs. This could be attributed to the fact that cells differ in ROS generation on subcellular levels. In macrophages, free radical generation in mitochondria is generally higher than in this case, which results in T1 values being closer to saturation and thus a lower variability. The mitochondria in cGC are taking part in a variety of free radical generating pathways, and thus free radical generation can differ a lot in ways from what activities they are involved. In contrast, free radical values in the cytoplasm are more stable probably due to less free radical-generating pathway involvement. In comparison, the distributions of T1 measured by bare-FND and aVDAC2-FND were rather comparable in mGCs, indicating a relatively equivalent variation of the free radical response between mitochondria and cytoplasm in mGCs.

There are several advantages of this technique. Relaxometry enables temporospatial measurements of the free radical load, which means the detection can be performed in real-time and on subcellular levels. To date, different methods, either indirect or direct, have been utilized in several studies measuring ROS levels in cumulus and/or mural granulosa cells.^15^ Indirect detection of ROS in biological samples by measuring ROS-induced lipid, protein and DNA modification is gaining popularity due to its stability and reliability.^13^ For example, markers of ROS-induced lipid modification such as malondialdehyde (MDA), which is based on the principle that free radicals induce lipid peroxidation by attacking lipids containing carbon–carbon double bonds, has been used in a recent study to investigate the correlation between cGC ROS and oocyte quality.^37^ Although these fluorescent dye-based methods have a high sample throughput within several minutes and appear stable and reliable in clinical settings, it is a one-time measurement not capable of detecting temporospatial changes upon oxidative stress induction. Additionally, this method serves as a bulk assay where information from many cells is averaged, and it is limited in spatial resolution, since dye molecules can diffuse freely in cells. It is also a potential drawback that no further treatment or analysis of that group of cells is possible. In the current study, two traditional assays (DCFH-DA and MitoSox) were applied for comparison to measure the cytoplasm and mitochondria. Also, the results should be cautiously interpreted due to photobleaching with time.^38^ In addition, to eliminate the background effects, a control group containing cells without treatment is always required, which means that the results are always a percentage change over the control group and are not suitable for basal ROS measurement across different patients. Further, due to different detected radicals and reaction principle, assay results from different kits cannot be compared directly, and thus, the main source of ROS production is hardly tracked. Finally, consistent to previous findings,^27^ our results show that free radical change occurs earlier than those in either of the two other methods, suggesting higher sensitivity of T1 measurements compared to these traditional methods. With respect to clinical practice, the single-cell resolution property of diamond relaxometry provides an ideal solution to ROS detection with high resolution in mGCs and cGCs, even with low cell numbers, and their association with oocyte competence and embryo development can be explored in assisted reproductive techniques.

However, quantum sensing also has points that should be approached cautiously. First, there is considerable variability among T1 values measured by different FNDs. This can be explained by differences between particles in size, shape, and exact surface area. In addition, nanodiamonds can be in a different environment within the cells, and thus free radical concentrations can vary within a few nanometers due to their short-life nature. However, since nanodiamonds allow long-term measurements, it is possible to follow a specific particle and thus differentiate between the original variability and changes due to the intervention, such as a menadione challenge in our case. The T1 changes induced by the oxidant are sensitive and robust, suggesting that this nanoscale MRI may perform better in the research of free radical change than detecting free radicals in bulk samples, where a large amount of cells and medium is measured at once. Second, diamond relaxometry requires a FND inside cells, which means the uptake of FNDs by cells is a premise of T1 measurement, and it takes some time for the FND to be taken up by cells and directed to the organelle of interest. However, this also applies to dye-based methods. Since with FNDs extremely small amounts (single particles) are needed for a measurement, they are usually tolerated better than conventional dyes. Additionally, it needs to be noted that our measurements are very local. This is an advantage for spatial resolution but might lead to relevant stress responses that we miss since they occur in a location that is not accessible for nanodiamonds. Finally, we conducted measurements in a specific cell type, but there might be other cell types that could be interesting to study as well.

In conclusion, this study demonstrates the feasibility of diamond relaxometry as a novel, sensitive method for measuring free radical changes in human granulosa cells in real-time and at subcellular levels. In addition, time-dependent free radical generation in response to oxidants differs on a subcellular level as well as between the cumulus and granulosa cells. Studies of cGC and mGC free radical in patients of different infertility factors can be expected by using this diamond relaxometry technique and would be interesting and additive to the current work.

## Methods

### Patient Inclusion and Sample Collection

Primary granulosa cells were obtained from preovulatory follicles from individual healthy women between 20 and 35 years old undergoing ovum pick-up for *in vitro* fertilization (IVF) at the department of Reproductive Medicine University Medical Centre Groningen, The Netherlands from October 2022 to February 2023. Inclusion criteria were as follows: (1) 25–35 years old; (2) normal menstrual cycle; (3) standard long hyperstimulation protocol; (4) at least 3 follicles with diameter >18 mm at the day of follicle triggering; (5) intracytoplasmic sperm injection (ICSI). Women with polycystic ovarian syndrome, endometriosis, diminished ovarian reserve, chromosome abnormality, or hydrosalpinx were excluded since these ovarian factors might affect follicle growth, and granulosa cells may thus behave very differently under oxidative stress.

Ethical approval from the Institutional Review Board was requested and waived since anonymized waste material (granulosa cells that routinely become available after oocyte retrieval) was used. An informed consent form was signed by all patients, and their material was processed anonymously. (All patients agreed on the use of their cumulus granulosa cells and mural granulosa cells, which routinely become available after oocyte retrieval and otherwise would be discarded as waste material.)

Generally, during the use of oral contraceptive pills (OCP) hormonal downregulation was started with daily injections of subcutaneous triptorelin 0.5 mg of GnRH analogue mg (Decapeptyl, Ferring Pharmaceuticals, The Netherlands). After 12 days, patients received human menopausal gonadotrophin 150–225 international unit (IU) per day (Menopur, Ferring Pharmaceuticals,The Netherlands) or Follitropine alfa rec FSH 150–225 IU (Gonal F, Merck Serono, Italy). Oocytes were collected 36 h after injection of 250 μg of recombinant human chorionic gonadotropin (hCG) (Ovitrelle Merck Serono, Italy). During oocyte retrieval, follicular fluid containing mGCs was collected in 50 mL centrifuge tubes. Parts of the cumulus cell clusters were mechanically separated from the cumulus-oocyte complex and stored in 15 mL centrifuge tubes in a G-MPOS (Vitrolife). Samples of cGCs and mGCs were then brought to a cell culture hood for further purification and culture.

### Cell Isolation and Culture

The isolation of cGCs and mGCs was performed as previously described.^39^ In brief, cGC clusters were dispersed by gently pipetting before centrifugation in HBSS (Life technologies, USA) for 4 min at 1400 rpm. For mGCs isolation, the follicular fluid was centrifuged 7 min at 600*g*, and the pellet was resuspended in phosphate-buffered saline PBS. Blood cells were removed by layering the cell pellet using a 40% Percoll gradient (Fisher Scientific, cat. no. 10607095) and 20 min centrifugation at 600 g. Cells from the interface were collected and washed in PBS through 4 min centrifugation at 1400 rpm, followed by resuspension of pelleted cells in 1 mL of trypsin and 3 min incubation at 37 °C and pipetting to disperse the clustered cells. Both types of cells were then passed through a Falcon 40 μM strainer (Corner, cat. no. 352340), followed by being cultured in Dulbecco’s Modified Eagle Medium/Nutrient Mixture F-12 (DMEM/F12) (Life Technologies, USA, cat.no. 11320033) supplemented with 10% FCS, 1% penicillin-streptomycin-amphotericin B at 37 °C, and 5% CO_2_. For T1 measurements and confocal microscopy, cells were plated in 35 mm culture dishes (CELLview Culture dish, nontreated, 4 compartments, glass bottom, Greiner Bio-One) at a density of 4000 cells/compartment. For DCFHDA, MTT, and MitoSOX assays, cells were plated into culture at a density of 10000 cells/well of 96-well plates. After 48–72 h, the culture medium was refreshed. Cells were used in experiments within 1 week. The purity of cGCs and mGCs was confirmed by FSHR fluorescence microscopy.

### Materials

FNDs with a hydrodynamic diameter of 70 nm containing >300 NV centers purchased from Adámas Nanotechnologies (Raleigh, NC, USA) were selected since they are suitable for T1 measurement for several reasons.^24^ First, it takes a longer time for FNDs with smaller diameters to obtain a good signal-to-noise ratio due to their lower brightness. Second, it is comparatively more difficult to track smaller FNDs since they move faster. Larger particles also have the advantage that they contain more NV centers, and the measurement is already an average of all these NV centers, which greatly improved reproducibility. However, NV centers in the core of these larger particles are too distant from the surface to sense the spin noise from radicals. The particles we used are produced by high pressure and high temperature synthesis followed by grinding and size separation to the desired size. To increase the NV center yield nanodiamonds are irradiated with 3 MeV electrons at a fluence of 5 × 10^19^ e/cm^2^ and annealed at the temperature exceeding 700 °C by the manufacturer.^40^ As a last step of their synthesis by the manufacturer, FNDs are cleaned in oxidizing acid, resulting in oxygen terminated particles. These are widely used in the field and have been characterized before.^41^ Anti-VDAC2 antibody ([C2C3], C-term, catalog no. GTX104745) was obtained from GeneTex (The Netherlands). aVDAC2-FNDs were freshly prepared before use as previously described,^3^ followed by size and zeta potential measurements using the Malvern ZetaSizer Nanosystem (Dynamic Light Scattering; Malvern Instruments Ltd., Malvern, UK; www.malvern.com). Menadione (cat. no. M5625-25G) was purchased from Merck. Tom20 antibody (rabbit, catalog no. sc-11415) was purchased from Santa Cruz Biotechnology (USA). Goat-α-rabbit Alexa 405 secondary antibody (cat. No. A-31556) and MitoSOX Red mitochondrial superoxide indicator (cat. no. M36008) were bought from Thermo Fisher. Phalloidin–fluorescein isothiocyanate (FITC) was obtained from Sigma-Aldrich, The Netherlands. A cellular ROS assay kit (DCFDA/H2DCFDA, ab113851) was purchased from Abcam. FSH receptor Polyclonal Antibody (rabbit, cat. no. bs-0895R) was bought from Bioss (USA).

### Flow Cytometry

After isolation, both kinds of granulosa cells were separately fixed with 3.7% paraformaldehyde rather than being plated. Then cells were first incubated with rabbit-FSHR antibody diluted in 500 μL of 0.1% BSA for 3 h at room temperature, followed by incubation with 1:200 of goat-α-rabbit Alexa 488 secondary antibody for 45 min at room temperature protected from light. Cells without any staining were regarded as negative control groups. Cells stained with only a secondary antibody were also set to exclude unspecific binding. mGC samples before Percoll purification were also set for comparison. Cells were subjected to Quanteon Flow Cytometer Systems (Agilent Technologies, US) using a laser at 488 nm. Data acquisition and analyses were performed using NovoExpress software and gated for a high level of FITC expression.

### DCFDA Assay

cGCs and mGCs were seeded as 6 × 10^5^ per well in a 96-well cell culture plate (Tissue Culture-Treated, Flat-Bottom with lid, Corning) and incubated for 48 h to allow attaching to the bottom. For intracellular ROS measurement, cells were first washed with PBS and then incubated with 10 μg/mL DCFDA prevented from light at 37 °C for 45 min. Then the DCFDA staining solution was removed, and cells were washed and replaced with PBS. Next, cells were either treated with 10 μM menadione and the fluorescence intensity was measured. As a control we used cells with PBS only. As experimental groups, 5 time points were evaluated: 1, 5, 10, 20, and 30 min after menadione treatment. For experiments in which menadione served as a positive control of intracellular ROS induction, cells were first incubated with 5 μM menadione in 37 °C for 24 h before 10 μg/mL DCFDA was added and incubated at 37 °C for 45 min. All the fluorescence intensities were measured by a plate reader (Bio Tek, Santa Clara, CA) at Ex/Em = 485/535 nm prevented from light. Cells without staining with DCFDA were recorded for background subtraction.

### Mitochondrial Superoxide Detection

Mitochondrial superoxide was detected using the MitoSOX Mitochondrial Superoxide Indicator (Introgen, M36008) following the manufacturer’s protocol. Briefly, all samples except the control groups were treated with 10 μM menadione, and the medium was removed after 1, 5, 10, 20, and 30 min. After washing with PBS, 1 μM of the superoxide detection compound was added followed by incubation for 30 min at 37 °C. After staining, cells were washed in their cell culture medium twice, and the fluorescent product was measured by a plate reader (Bio Tek, Santa Clara, CA) at an excitation of 396 nm and an emission of 610 nm.

### MTT Assay

An MTT assay was carried out to evaluate the viabilities of cGCs and mGCs following nanodiamond incubation as well as determining a safe menadione concentration, which does not affect cell viability after incubation for 30 min. This assay provides an evaluation of cell metabolic activity by detecting nicotinamide adenine dinucleotide phosphate (NAD(P)H) dependent oxidoreductases activity. Cells cultured in 96-well plates were treated with 0.75 μg/mL MTT dissolved in DMEM/F12 medium. After 3 h of incubation at 37 °C, the reagent was removed, and 2-propanol was added to the samples to dissolve the formazan formed inside the cells. To confirm that FNDs do not affect cell viabilities, both kinds of cells were incubated with FNDs (1 and 5 μg/mL) for 24 h. To find a safe menadione concentration for cGC and mGC viability, both kinds of cells were pretreated by menadione of 2, 10, 50, 100 μM for 30 min. In all experiments, cGCs and mGCs treated with 0.1 M HCl were used as positive controls, while cells without any treatment were used as a negative control. The absorbance of the colored solution was measured by using a plate reader (Bio Tek, Santa Clara, CA) at 570 nm. All experiments were performed in six replicates. Samples were normalized against the mean absorbance value of the negative control, represented as a line at the value 1.

### Confocal Microscopy

For FND uptake experiments, cGCs and mGCs were incubated at 37 °C and 5% CO_2_ with both bare-FNDs and aVDAC2-FNDs (1 μg/mL) for 2 and 24 h, respectively. At each time point, the cell culture medium with FNDs was removed. After washing with 1× phosphate-buffered saline (PBS), cells were fixed with 3.7% paraformaldehyde for 10 min at room temperature. After fixation, cells were either covered with PBS and stored in at 4 °C for later staining or immediately stained. For FND uptake experiments, cells were first treated with 1% Triton X-100 for 3 min to permeabilize the cell membranes. Next, 5% PBSA was applied and incubated for 30 min to block the nonspecific background, followed by adding staining solution mixed by 4 μg/mL 4′,6-diamidino-2-phenylindole (DAPI) and 2 μg/mL phalloidin–fluorescein isothiocyanate (FITC) in PBSA for visualization of nuclei and F-actin, respectively. Finally, 500 μL of PBS was added to cover the sample for imaging. Images were taken using a 63× 1.30 GLYCEROL objective in a Leica SP8X DLS confocal microscope (Leica microsystems, Wetzlar, Germany) with a 405 nm laser to detect DAPI, a 488 nm laser to measure phalloidin-FITC, and a 561 nm laser to detect FNDs. Z-stacks were performed to determine the numbers of particles inside the cells at both time points. Three independent experiments were performed, and at least 30 cells were quantified for each time point.

For colocalization experiments, cGCs and mGCs were incubated at 37 °C and 5% CO_2_ with both bare-FNDs and aVDAC2-FNDs (1 μg/mL) for 24 h. After fixation, cell membrane permeation, and nonspecific background blockage as described above, cells were first incubated with rabbit-Tom20 antibody diluted in 500 μL of 0.1% BSA for 3 h at room temperature or overnight (1:500) at 4 °C. Then, cells were incubated with 1:200 of goat-α-rabbit Alexa 405 secondary antibody and 2 μg/mL phalloidin–FITC in 500 μL of 0.1% BSA for 45 min at room temperature protected from light. Finally, 500 μL of PBS was added to cover the samples for imaging. Images were taken using a 63× 1.30 GLYCEROL objective in a Leica SP8X DLS confocal microscope (Leica microsystems, Wetzlar, Germany) with a 405 nm laser to detect TOM20, a 488 nm laser to measure phalloidin-FITC, and a 561 nm laser to detect FNDs. All images were processed by using the FIJI software.

### T1 Measurements

Cells were first washed with PBS, and then PBS was replaced with DMEM/F12 medium after incubation with 1 μg/mL bare-FNDs or aVDAC2-FNDs for 24 h. After particle identification and localization, we performed T1 (relaxometry) measurements using a laser pulsing sequence in a custom-made magnetometry setup, which is in principle a confocal microscope with some modifications^42^ (Figure 1C).

In a typical T1 measurement, we pump NV centers into the bright ms = 0 state of the ground state. We then probed after different dark times if the NV centers remained in this state or returned to the darker equilibrium between ms = 0 and ms = + −1. In the presence of free radicals this process occurs faster and can be used to quantify free radical generation.^43^ To extract the magnetic noise level from these plots, we used a double exponential fit of the form:
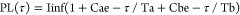
1This fit is different from the single exponential fits that are used for single NV center measurements. After the observation was made that the single exponential model does not represent the data well in ensembles, this model was determined empirically. This fit considers that there are different NV centers with different T1 values within each particle. While both constants respond to changes in magnetic noise, the longer constant is more sensitive to changes in magnetic noise. This was found earlier by measuring different known concentrations and observing how the different constants respond. Thus, to quantify free radical generation we use the longer time constant Tb which we call T1.^24^ A detailed discussion of the biexponential model as well as comparison with other models can be found.^44^

This measurement reveals a signal that is equivalent to T1 in conventional MRI. However, since NV centers only detect their local environment (up to a few tens of nm), this method offers nanoscale resolution.^45^

The laser we used is a 532 nm laser at 50 μW at the location of the sample (measured in continuous illumination). The measurement sequence consisted of 5 μs long laser pulses separated by variable dark times τ from 0.2 to 1000 μs. To conduct the pulsing sequence, an acousto-optical modulator (Gooch & Housego, model 3350-199) and a magnification oil objective (×100) (Olympus, UPLSAPO 100XO, NA 1.40) were applied. Under a bright field camera (Thorlabs), the following criteria were checked and confirmed before an FND particle was selected: 1. The FND was well located inside a cell; 2. the brightness was around 3 million photon counts/s; 3. The fluorescence is stable since bleaching structures are background fluorescence rather than FNDs. Then, the first T1 measurement of the selected FND was performed. After this measurement, menadione (10 μM) was gently added to the DMEM/F12 medium to trigger free radical generation. T1 measurements on the same FND were recorded at 1, 5, 10, 20, 30 min after menadione addition. Before each measurement, it was again confirmed that a particle was an FND by tracking its location, photon count, and stable fluorescence. All T1 measurements were conducted at room temperature and under ambient air. Due to the relatively low laser power and the fact that the laser is mostly off during a T1 measurement, we did not observe any measurable heating.^26^

### Statistical Analysis

Quantitative data were presented as the mean ± standard deviation (SD). All statistical tests were conducted using R programming language. Significance was tested by using one-way ANOVA followed by a Tukey post hoc test or *t* test and is specifically indicated in the legend of each figure. All statistical tests were compared to the control group and defined as ns *P* > 0.05, *P ≤ 0.05, **P ≤ 0.01, ***P ≤ 0.001, and ****P ≤ 0.0001.

## Data Availability

The data used to support the finding of this study are available from the corresponding author upon request.

## Supporting Information Available

Size and zeta potential of bare-FNDs and aVDAC2-FNDs; identification of human primary cumulus granulosa cells and their purity by flow cytometry; confocal images to confirm nanodiamond uptake; nanodiamond quantification; measurements of cell viability after menadione treatment and in the presence of FNDs; comparison with conventional ROS detection assay; control T1 measurements of cells without intervention and nanodiamonds with menadione in absence of cells; raw data from T1 experiments with cells treated with Menadione and control cells (PDF)
Transparent Peer Review report available (PDF)

## References

[ref1] Eppig J. J.; Chesnel F.; Hirao Y.; O’Brien M. J.; Pendola F. L.; Watanabe S.; Wigglesworth K. Oocyte control of granulosa cell development: how and why. Hum. Reprod. 1997, 12 (11 Suppl), 127–132. 9433969

[ref2] Li R.; Norman R. J.; Armstrong D. T.; Gilchrist R. B. Oocyte-secreted factor(s) determine functional differences between bovine mural granulosa cells and cumulus cells. Biol. Reprod. 2000, 63 (3), 839–845. 10.1095/biolreprod63.3.839. 10952929

[ref3] Wigglesworth K.; Lee K. B.; Emori C.; Sugiura K.; Eppig J. J. Transcriptomic diversification of developing cumulus and mural granulosa cells in mouse ovarian follicles. Biol. Reprod. 2015, 92 (1), 23 10.1095/biolreprod.114.121756. 25376232 PMC4434932

[ref4] Koks S.; Velthut A.; Sarapik A.; Altmae S.; Reinmaa E.; Schalkwyk L.C.; Fernandes C.; Lad H.V.; Soomets U.; Jaakma U.; Salumets A.; et al. The differential transcriptome and ontology profiles of floating and cumulus granulosa cells in stimulated human antral follicles. Mol. Hum. Reprod. 2010, 16 (4), 229–240. 10.1093/molehr/gap103. 19933312

[ref5] Agarwal A.; Aponte-Mellado A.; Premkumar B. J.; Shaman A.; Gupta S. The effects of oxidative stress on female reproduction: a review. Reprod Biol. Endocrinol 2012, 10, 49 10.1186/1477-7827-10-49. 22748101 PMC3527168

[ref6] Shkolnik K.; Tadmor A.; Ben-Dor S.; Nevo N.; Galiani D.; Dekel N. Reactive oxygen species are indispensable in ovulation. Proc. Natl. Acad. Sci. U. S. A. 2011, 108 (4), 1462–1467. 10.1073/pnas.1017213108. 21220312 PMC3029775

[ref7] Hu Y.; Betzendahl I.; Cortvrindt R.; Smitz J.; Eichenlaub-Ritter U. Effects of low O2 and ageing on spindles and chromosomes in mouse oocytes from pre-antral follicle culture. Hum. Reprod. 2001, 16 (4), 737–748. 10.1093/humrep/16.4.737. 11278227

[ref8] Combelles C. M.; Gupta S.; Agarwal A. Could oxidative stress influence the in-vitro maturation of oocytes?. Reprod Biomed Online 2009, 18 (6), 864–880. 10.1016/S1472-6483(10)60038-7. 19490793 PMC3235363

[ref9] Pasqualotto E. B.; Agarwal A.; Sharma R. K.; Izzo V. M.; Pinotti J. A.; Joshi N. J.; Rose B. I. Effect of oxidative stress in follicular fluid on the outcome of assisted reproductive procedures. Fertil Steril 2004, 81 (4), 973–976. 10.1016/j.fertnstert.2003.11.021. 15066450

[ref10] Desai N.; Sabanegh E. Jr.; Kim T.; Agarwal A. Free radical theory of aging: implications in male infertility. Urology 2010, 75 (1), 14–19. 10.1016/j.urology.2009.05.025. 19616285

[ref11] Sasaki H.; Hamatani T.; Kamijo S.; Iwai M.; Kobanawa M.; Ogawa S.; Miyado K.; Tanaka M. Impact of Oxidative Stress on Age-Associated Decline in Oocyte Developmental Competence. Front Endocrinol (Lausanne) 2019, 10, 811 10.3389/fendo.2019.00811. 31824426 PMC6882737

[ref12] Jiang J. Y.; Xiong H.; Cao M.; Xia X.; Sirard M. A.; Tsang B. K. Mural granulosa cell gene expression associated with oocyte developmental competence. J. Ovarian Res. 2010, 3, 6 10.1186/1757-2215-3-6. 20205929 PMC2845131

[ref13] Murphy M. P.; Bayir H.; Belousov V.; Chang C. J.; Davies K. J. A.; Davies M. J.; Dick T. P.; Finkel T.; Forman H. J.; Janssen-Heininger Y.; et al. Guidelines for measuring reactive oxygen species and oxidative damage in cells and in vivo. Nat. Metab 2022, 4 (6), 651–662. 10.1038/s42255-022-00591-z. 35760871 PMC9711940

[ref14] He W.; Liu Y.; Wamer W. G.; Yin J. J. Electron spin resonance spectroscopy for the study of nanomaterial-mediated generation of reactive oxygen species. J. Food Drug Anal 2014, 22 (1), 49–63. 10.1016/j.jfda.2014.01.004. 24673903 PMC9359146

[ref15] Seino T.; Saito H.; Kaneko T.; Takahashi T.; Kawachiya S.; Kurachi H. Eight-hydroxy-2’-deoxyguanosine in granulosa cells is correlated with the quality of oocytes and embryos in an in vitro fertilization-embryo transfer program. Fertil Steril 2002, 77 (6), 1184–1190. 10.1016/S0015-0282(02)03103-5. 12057726

[ref16] Sharmin R.; Nusantara A. C.; Nie L.; Wu K.; Elias Llumbet A.; Woudstra W.; Mzyk A.; Schirhagl R. Intracellular Quantum Sensing of Free-Radical Generation Induced by Acetaminophen (APAP) in the Cytosol, in Mitochondria and the Nucleus of Macrophages. ACS Sens 2022, 7 (11), 3326–3334. 10.1021/acssensors.2c01272. 36354956 PMC9706807

[ref17] Hsiao W. W.; Hui Y. Y.; Tsai P. C.; Chang H. C. Fluorescent Nanodiamond: A Versatile Tool for Long-Term Cell Tracking, Super-Resolution Imaging, and Nanoscale Temperature Sensing. Acc. Chem. Res. 2016, 49 (3), 400–407. 10.1021/acs.accounts.5b00484. 26882283

[ref18] Panwar N.; Soehartono A. M.; Chan K. K.; Zeng S.; Xu G.; Qu J.; Coquet P.; Yong K. T.; Chen X. Nanocarbons for Biology and Medicine: Sensing, Imaging, and Drug Delivery. Chem. Rev. 2019, 119 (16), 9559–9656. 10.1021/acs.chemrev.9b00099. 31287663

[ref19] Schirhagl R.; Chang K.; Loretz M.; Degen C. L. Nitrogen-vacancy centers in diamond: nanoscale sensors for physics and biology. Annu. Rev. Phys. Chem. 2014, 65, 83–105. 10.1146/annurev-physchem-040513-103659. 24274702

[ref20] Žurauskas M.; Alex A.; Park J.; Hood S. R.; Boppart S. A. Fluorescent nanodiamonds for characterization of nonlinear microscopy systems. Photonics Res. 2021, 9 (12), 2309–2318. 10.1364/PRJ.434236. 37181134 PMC10174270

[ref21] Neumann P.; Jakobi I.; Dolde F.; Burk C.; Reuter R.; Waldherr G.; Honert J.; Wolf T.; Brunner A.; Shim J. H.; Suter D.; Sumiya H.; Isoya J.; Wrachtrup J. High-precision nanoscale temperature sensing using single defects in diamond. Nano Lett. 2013, 13 (6), 2738–2742. 10.1021/nl401216y. 23721106

[ref22] Yip K. Y.; Ho K. O.; Yu K. Y.; Chen Y.; Zhang W.; Kasahara S.; Mizukami Y.; Shibauchi T.; Matsuda Y.; Goh S. K.; et al. Measuring magnetic field texture in correlated electron systems under extreme conditions. Science 2019, 366 (6471), 1355–1359. 10.1126/science.aaw4278. 31831663

[ref23] Thiel L.; Wang Z.; Tschudin M. A.; Rohner D.; Gutiérrez-Lezama I.; Ubrig N.; Gibertini M.; Giannini E.; Morpurgo A. F.; Maletinsky P. J. S. Probing magnetism in 2D materials at the nanoscale with single-spin microscopy. Science 2019, 364 (6444), 973–976. 10.1126/science.aav6926. 31023891

[ref24] Perona Martínez F.; Nusantara A. C.; Chipaux M.; Padamati S. K.; Schirhagl R. Nanodiamond Relaxometry-Based Detection of Free-Radical Species When Produced in Chemical Reactions in Biologically Relevant Conditions. ACS Sens 2020, 5 (12), 3862–3869. 10.1021/acssensors.0c01037. 33269596 PMC8651177

[ref25] Reyes-San-Martin C.; Hamoh T.; Zhang Y.; Berendse L.; Klijn C.; Li R.; Llumbet A. E.; Sigaeva A.; Kawałko J.; Mzyk A.; et al. Nanoscale MRI for Selective Labeling and Localized Free Radical Measurements in the Acrosomes of Single Sperm Cells. ACS Nano 2022, 16 (7), 10701–10710. 10.1021/acsnano.2c02511. 35771989 PMC9331174

[ref26] Nie L.; Nusantara A. C.; Damle V. G.; Sharmin R.; Evans E. P. P.; Hemelaar S. R.; van der Laan K. J.; Li R.; Perona Martinez F. P.; Vedelaar T.; Chipaux M.; Schirhagl R. Quantum monitoring of cellular metabolic activities in single mitochondria. Sci. Adv. 2021, 7 (21), eabf0573 10.1126/sciadv.abf0573. 34138746 PMC8133708

[ref27] Wu K.; Nie L.; Nusantara A. C.; Woudstra W.; Vedelaar T.; Sigaeva A.; Schirhagl R. Diamond Relaxometry as a Tool to Investigate the Free Radical Dialogue between Macrophages and Bacteria. ACS Nano 2023, 17 (2), 1100–1111. 10.1021/acsnano.2c08190. 36630151 PMC9878971

[ref28] Morita A.; Nusantara A. C.; Myzk A.; Perona Martinez F. P.; Hamoh T.; Damle V. G.; van der Laan K. J.; Sigaeva A.; Vedelaar T.; Chang M.; et al. Detecting the metabolism of individual yeast mutant strain cells when aged, stressed or treated with antioxidants with diamond magnetometry. Nano Today 2023, 48, 101704 10.1016/j.nantod.2022.101704.

[ref29] Simoni M.; Gromoll J.; Nieschlag E. The follicle-stimulating hormone receptor: biochemistry, molecular biology, physiology, and pathophysiology. Endocr Rev. 1997, 18 (6), 739–773. 10.1210/edrv.18.6.0320. 9408742

[ref30] Fukui M.; Choi H. J.; Zhu B. T. Rapid generation of mitochondrial superoxide induces mitochondrion-dependent but caspase-independent cell death in hippocampal neuronal cells that morphologically resembles necroptosis. Toxicol. Appl. Pharmacol. 2012, 262 (2), 156–166. 10.1016/j.taap.2012.04.030. 22575170 PMC3748803

[ref31] Criddle D. N.; Gillies S.; Baumgartner-Wilson H. K.; Jaffar M.; Chinje E. C.; Passmore S.; Chvanov M.; Barrow S.; Gerasimenko O. V.; Tepikin A. V.; et al. Menadione-induced reactive oxygen species generation via redox cycling promotes apoptosis of murine pancreatic acinar cells. J. Biol. Chem. 2006, 281 (52), 40485–40492. 10.1074/jbc.M607704200. 17088248

[ref32] Nohl H.; Gille L. Lysosomal ROS formation. Redox Rep 2005, 10 (4), 199–205. 10.1179/135100005X70170. 16259787

[ref33] Zorov D. B.; Juhaszova M.; Sollott S. J. Mitochondrial reactive oxygen species (ROS) and ROS-induced ROS release. Physiol Rev. 2014, 94 (3), 909–950. 10.1152/physrev.00026.2013. 24987008 PMC4101632

[ref34] Hoque S. A. M.; Kawai T.; Zhu Z.; Shimada M. Mitochondrial Protein Turnover Is Critical for Granulosa Cell Proliferation and Differentiation in Antral Follicles. J. Endocr Soc. 2019, 3 (2), 324–339. 10.1210/js.2018-00329. 30652133 PMC6330174

[ref35] Liu Y.; Han M.; Li X.; Wang H.; Ma M.; Zhang S.; Guo Y.; Wang S.; Wang Y.; Duan N. J. H. r. Age-related changes in the mitochondria of human mural granulosa cells. Hum. Reprod. 2017, 32 (12), 2465–2473. 10.1093/humrep/dex309. 29045673

[ref36] von Mengden L.; Klamt F.; Smitz J. Redox Biology of Human Cumulus Cells: Basic Concepts, Impact on Oocyte Quality, and Potential Clinical Use. Antioxid Redox Signal 2020, 32 (8), 522–535. 10.1089/ars.2019.7984. 31861967 PMC7038817

[ref37] Tural R.; Karakaya C.; Erdem M.; Aykol Z.; Karabacak R. O.; Kavutcu M. J. T. J. o. M. S. Investigation of oxidative stress status in cumulus cells in patıents with in vitro fertilization. Turk J Med Sci 2021, 51 (4), 1969–1975. 10.3906/sag-2104-188. 34344144

[ref38] Figueroa D.; Asaduzzaman M.; Young F. Real time monitoring and quantification of reactive oxygen species in breast cancer cell line MCF-7 by 2’,7’-dichlorofluorescin diacetate (DCFDA) assay. J. Pharmacol Toxicol Methods 2018, 94 (Pt 1), 26–33. 10.1016/j.vascn.2018.03.007. 29630935

[ref39] Nagy R. A.; Hollema H.; Andrei D.; Jurdzinski A.; Kuipers F.; Hoek A.; Tietge U. J. F. The Origin of Follicular Bile Acids in the Human Ovary. Am. J. Pathol. 2019, 189 (10), 2036–2045. 10.1016/j.ajpath.2019.06.011. 31369754

[ref40] Shenderova O. A.; Shames A. I.; Nunn N. A.; Torelli M. D.; Vlasov I.; Zaitsev A. Review Article: Synthesis, properties, and applications of fluorescent diamond particles. J. Vac Sci. Technol. B Nanotechnol Microelectron 2019, 37 (3), 030802 10.1116/1.5089898. 31032146 PMC6461556

[ref41] Su Z.; Ren Z.; Bao Y.; Lao X.; Zhang J.; Zhang J.; Zhu D.; Lu Y.; Hao Y.; Xu S. J. J. o. M. C. C. Luminescence landscapes of nitrogen-vacancy centers in diamond: quasi-localized vibrational resonances and selective coupling. J. Mater. Chem. 2019, 7 (26), 8086–8091.

[ref42] Wu K.; Vedelaar T. A.; Damle V. G.; Morita A.; Mougnaud J.; Reyes San Martin C.; Zhang Y.; van der Pol D. P. I.; Ende-Metselaar H.; Rodenhuis-Zybert I.; et al. Applying NV center-based quantum sensing to study intracellular free radical response upon viral infections. Redox Biol. 2022, 52, 102279 10.1016/j.redox.2022.102279. 35349928 PMC8965164

[ref43] Rollo M.; Finco A.; Tanos R.; Fabre F.; Devolder T.; Robert-Philip I.; Jacques V. J. P. R. B. Quantitative study of the response of a single NV defect in diamond to magnetic noise. Phys. Rev. B 2021, 103 (23), 235418.

[ref44] Vedelaar T. A.; Hamoh T. H.; Martinez F. P.; Chipaux M.; Schirhagl R. J. Optimising data processing for nanodiamond based relaxometry.arXiv 2022; arXiv:2211.07269, 10.48550/arXiv.2211.07269.

[ref45] Mamin H.; Kim M.; Sherwood M.; Rettner C.; Ohno K.; Awschalom D.; Rugar D. J. S. Nanoscale nuclear magnetic resonance with a nitrogen-vacancy spin sensor. Science 2013, 339 (6119), 557–560. 10.1126/science.1231540. 23372008

