# Supplementary material for: Quantum Sensing of Free Radicals in Primary Human Granulosa Cells with Nanoscale Resolution
Source: ACS Cent Sci. 2023 Aug 30;9(9):1784–98. doi: 10.1021/acscentsci.3c00747 (PMC10540281; doi:10.1021/acscentsci.3c00747)
Supplement: Supplementary file 1 — oc3c00747_si_001.pdf [file oc3c00747_si_001.pdf]

Supplementary information

Title: Quantum sensing of free radicals in primary human granulosa cells with nanoscale resolution

Nuan Lin[1,2], Koen van Zomeren[1], Teelkien van Veen[1], Aldona Mzyk[3,4], Yue Zhang[3], Xiaoling Zhou[5], Torsten Plosch[1], Uwe J. F. Tietge[6,7], Astrid Cantineau[1], Annemieke Hoek[1*], Romana Schirhagl[3*]

[1]Department of Obstetrics and Gynecology, University of Groningen, University Medical Center Groningen, Groningen, The Netherlands
[2]Department of Obstetrics and Gynecology, The First Affiliated Hospital of Shantou University Medical College, Shantou, China
[3]Department of Biomedical Engineering, Groningen University, University Medical Center Groningen, Antonius Deusinglaan 1, 9713 AW Groningen, The Netherlands
[4]Institute of Metallurgy and Materials Science, Polish Academy of Sciences, Reymonta 25, 30-059 Krakow, Poland.
[5]Center for Reproductive Medicine, Shantou University Medical College, Shantou 515041, China
[6]Division of Clinical Chemistry, Department of Laboratory Medicine, Karolinska Institutet, Stockholm, Sweden
[7]Clinical Chemistry, Karolinska University Laboratory, Karolinska University Hospital, Stockholm, SE-141 86 Stockholm, Sweden.
[*] Corresponding Authors

Romana Schirhagl – Groningen University, University Medical Center Groningen, 9713 AW Groningen, The Netherlands; orcid.org/0000-0002-8749-1054; Email: romana.schirhagl@gmail.com

Annemieke Hoek – University of Groningen, University Medical Center Groningen, Groningen, The Netherlands, Email: a.hoek@umcg.nl

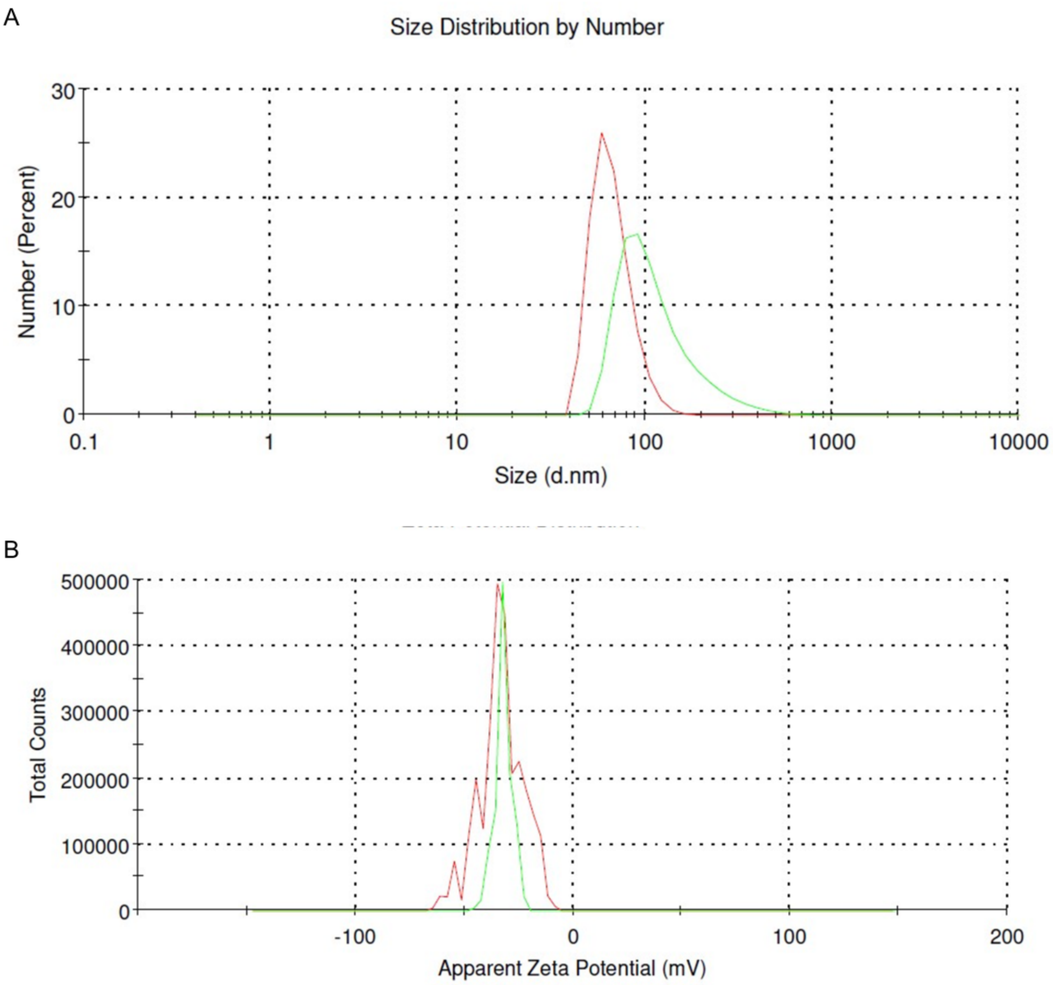

Figure S1. Size and zeta potential of bare-FNDs and aVDAC2-FNDs measured by the Malvern ZetaSizer Nano system. The size of uncoated FNDs (orange) is 89.28 nm (PDI = 0.053) and zeta potential is -33mV. For aVDAC2-FND (green), size is 222 nm in size (poly-dispersity index (PDI) is 0.187) and zeta potential is -32mV.

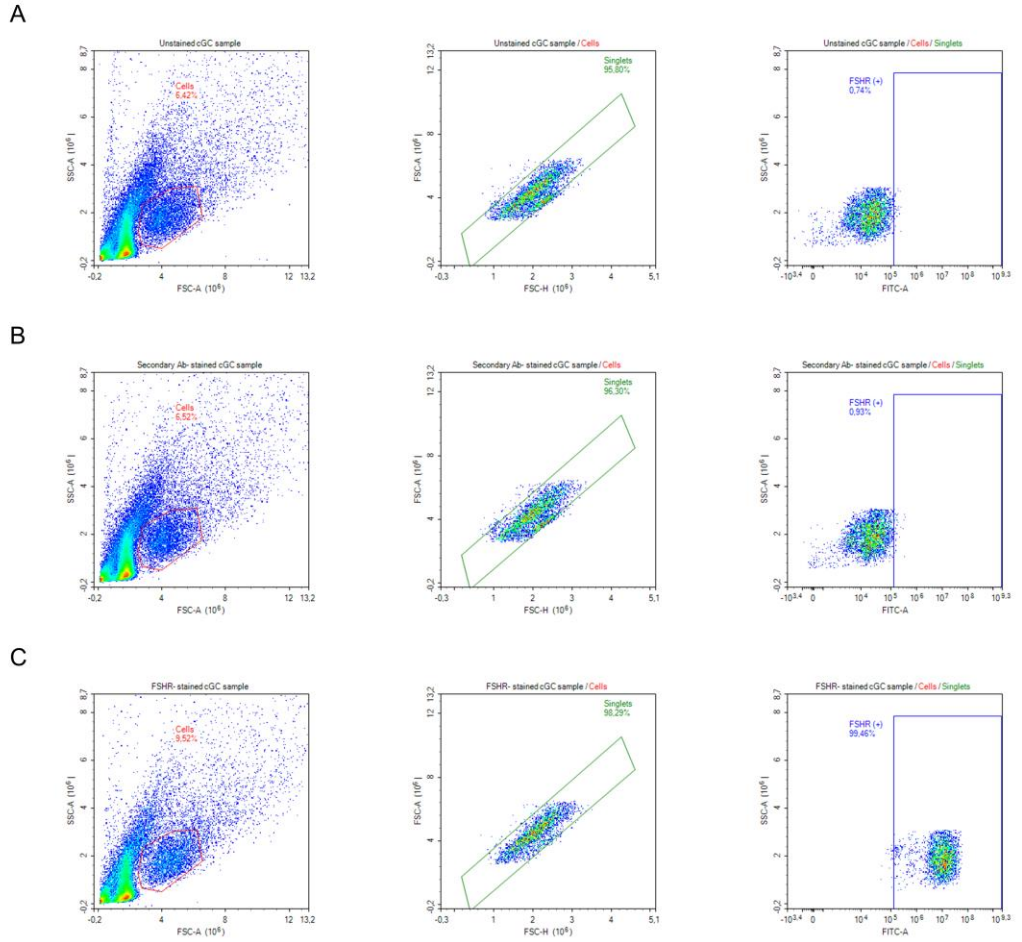

Figure S2. Identification of human primary cumulus granulosa cells (cGCs). Samples were gated to exclude debris, doublets before identifying FSHR+ subsets in (A) unstained cGC sample; (B) secondary antibody stained cGC sample to exclude unspecific binding of secondary antibody (C) FSHR and secondary antibody stained cGC sample.

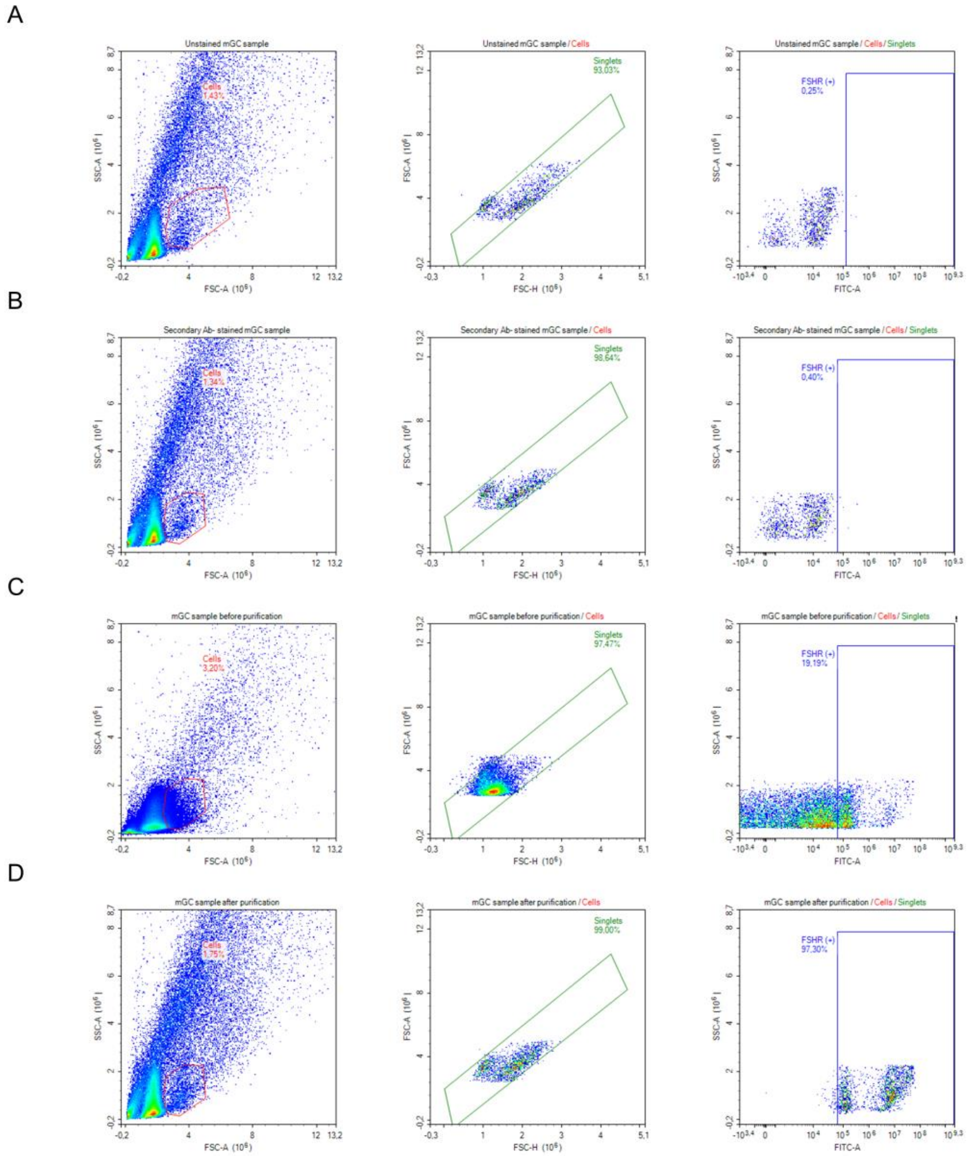

Figure S3. Identification of human primary mural granulosa cells (mGCs). Samples were gated to exclude debris, doublets before identifying FSHR+ subsets in (A) unstained mGC sample; (B) secondary antibody stained mGC sample to exclude unspecific binding of secondary antibody; (C) FSHR and secondary antibody stained mGC sample before purification; (D) FSHR and secondary antibody stained mGC sample after purification.

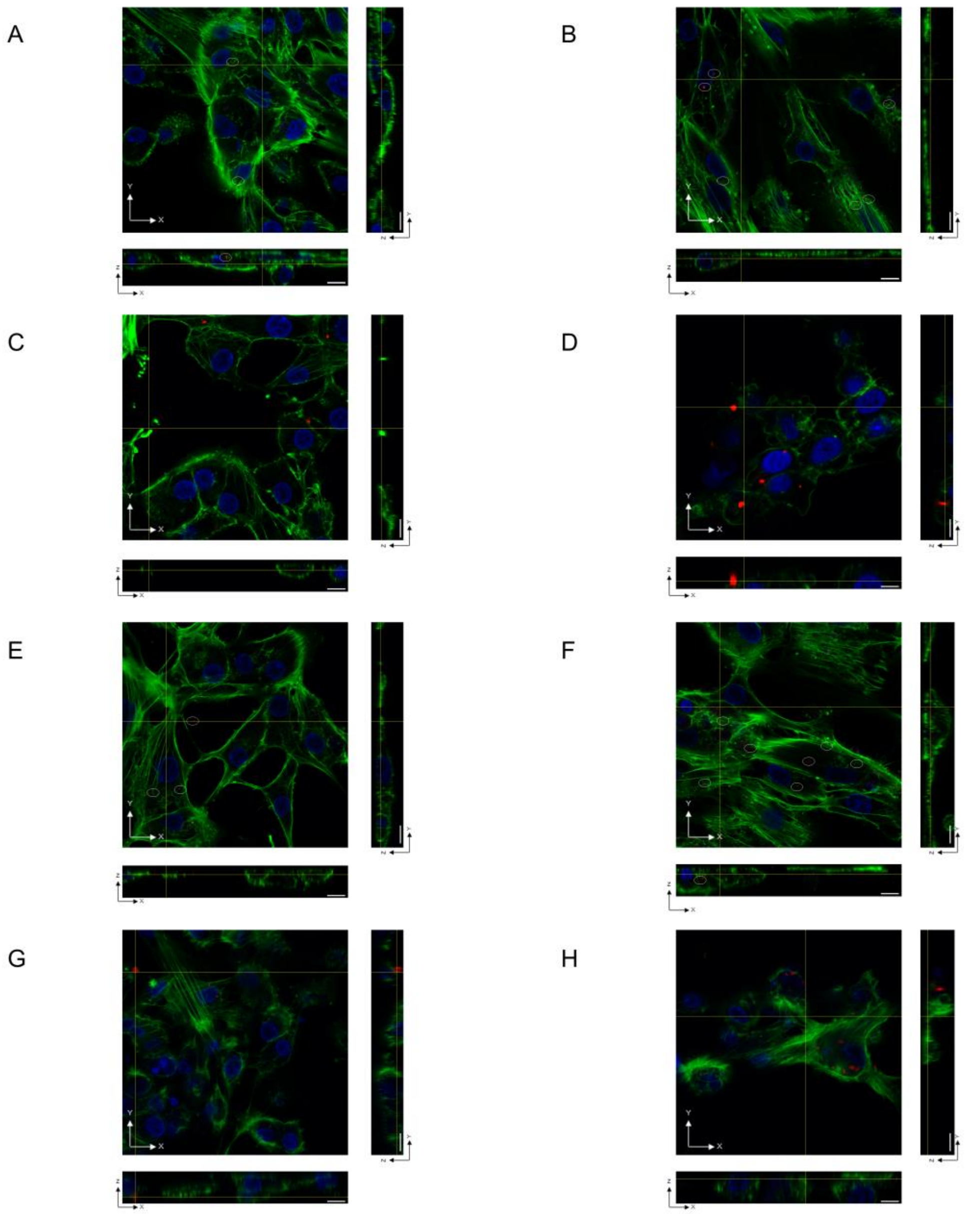

Figure S4. Confocal fluorescence microscopy shows orthogonal view images (xy, xz, and yz scans) of the diamond uptake by primary human granulosa cell. cGCs incubated with 1 µg/mL bare FNDs for 2 hours (A) and 24 hours (B); cGCs incubated with 1 µg/mL aVDAC2-FNDs for 2 hours (C) and 24 hours (D); mGCs incubated with 1 µg/mL bare FNDs for 2 hours (E) and 24 hours (F); mGCs incubated with 1 µg/mL aVDAC2-FNDs for 2 hours (G) and 24 hours (H). All images are 2D projections of the maximum pixel intensity obtained from 3D (XYZ) sample scans. Color code: green, Phalloidin-FITC, staining actin filaments (also known as F-actin); blue: DAPI (staining DNA); red: bare-FNDs or aVDAC2-FNDs. Scale bar, 10 µm.

A

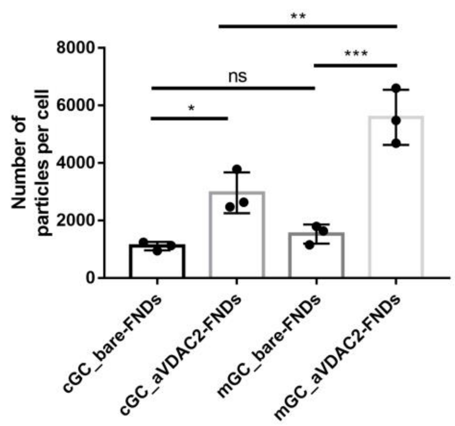

B

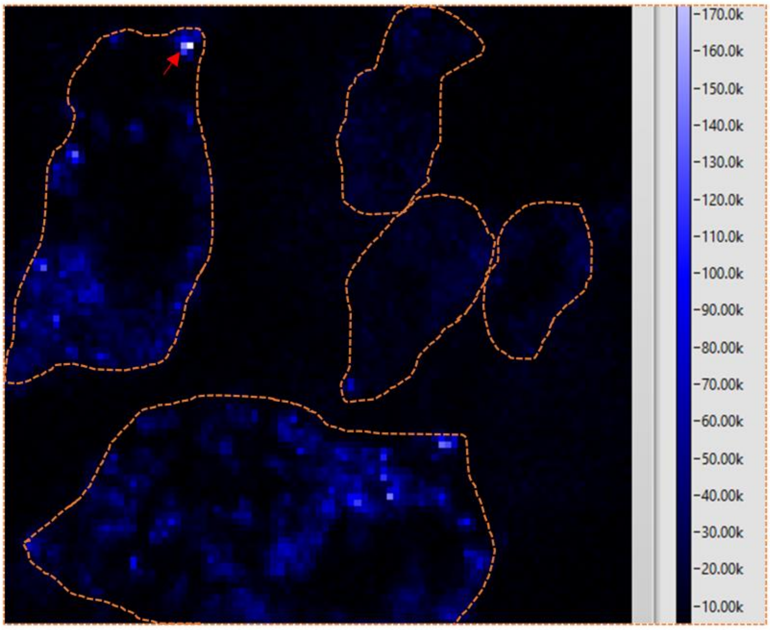

Figure S5. Diamond uptake after incubation for 24 hours. (A) Quantitative analysis of FND uptake per cell. For each group, the analysis was performed on cells from three different patients; at least 30 cells were counted for each patient sample. Significance was tested by using a one-way ANOVA followed by a Tukey post hoc test. ns $p>0.05$ * $p < 0.5$, ** $p < 0.01$, *** $p < 0.001$. (B) Representative fluorescence image of bare-FND in granulosa cells. The dashed line is the cell border. Inset: T1 relaxation measurement and refocus on the chosen FND particle during the measurement.

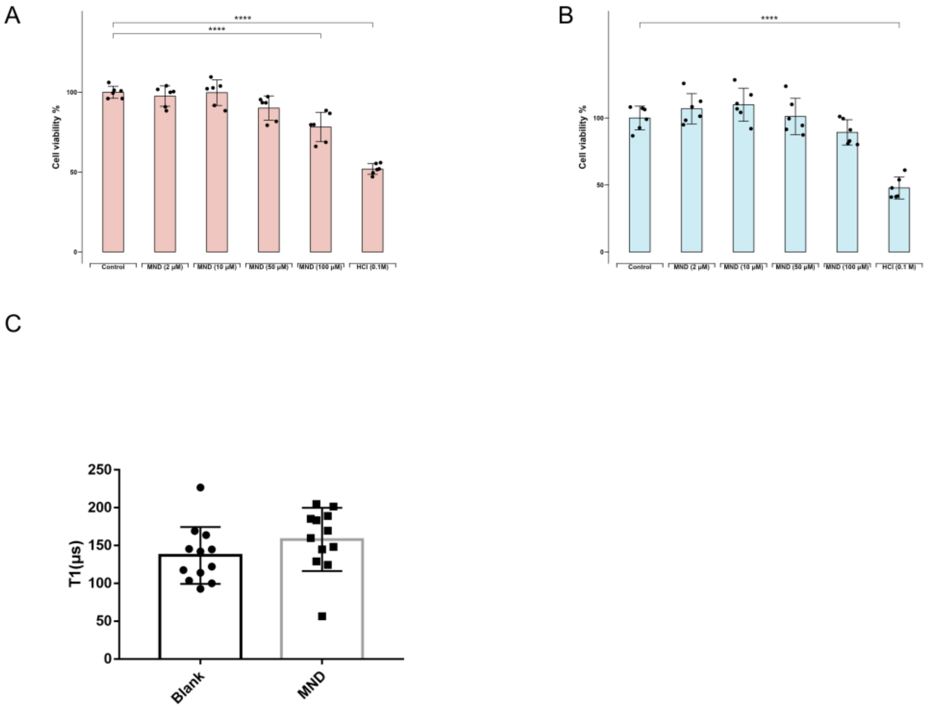

Figure S6. Effects of menadione of cell viability and per se T1 value. (A) MTT assay show cell viability followed by treatment of menadione of different concentration for 30 minutes in cGCs (A) and mGCs (B). (C) per se T1 value measured by bare-FNDs followed by 10 uM menadione in the absence of cells.

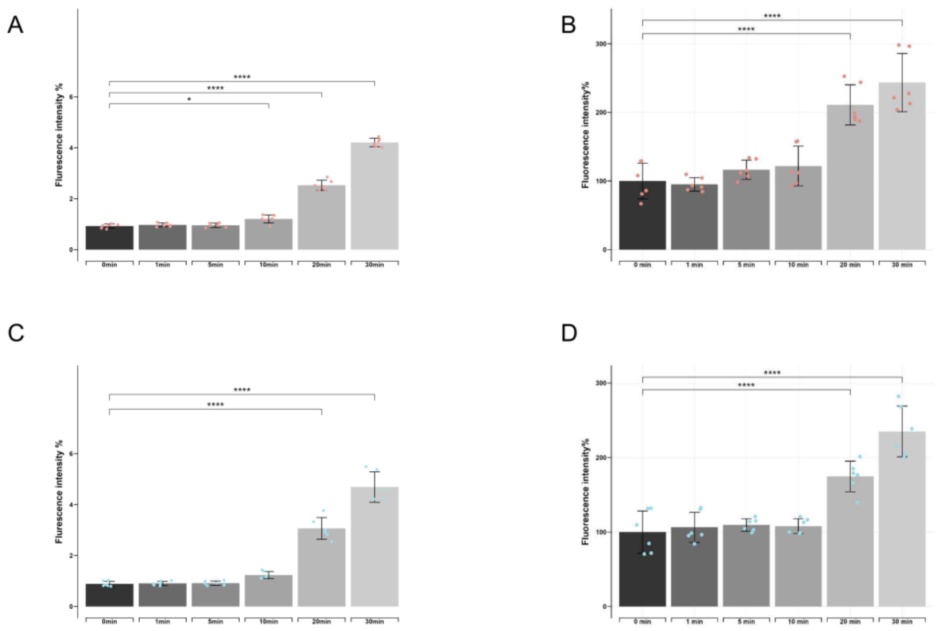

Figure S7. Intracellular reactive oxygen species (ROS) and mitochondrial superoxide following menadione treatment for different time duration measured by conventional methods. (A) Intracellular ROS in cGCs measured by 2,7-Dichlorodihydrofluorescein diacetate (DCFH-DA) assay kit; (B) mitochondrial superoxide levels in cGCs measured by Mitochondrial Superoxide Indicators (MitoSOX); (A) Intracellular ROS in mGCs measured by 2,7-Dichlorodihydrofluorescein diacetate (DCFH-DA) assay kit; (B) mitochondrial superoxide levels in mGCs measured by Mitochondrial Superoxide Indicators (MitoSOX). The data were analyzed by using one-way ANOVA followed by a Tukey post hoc test in comparison to the control groups. * $p < 0.5$, ** $p < 0.01$, *** $p < 0.001$, **** $p<0.0001$.

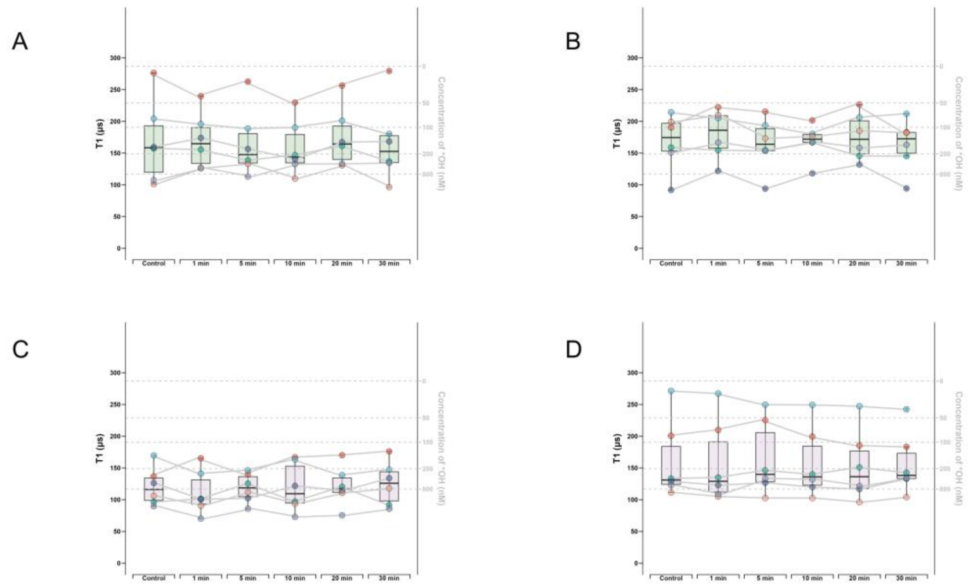

Figure S8. Box-whisker plots shows real-time free radical change followed by addition of DMSO-contained DMEM culture medium (no menadione) determined by T1 in granulosa cells at different time points. T1 of bare-FNDs from 4 patients in cGCs (A) and mGCs (B) were measured at 0, 1, 5, 10, 20, 30 minutes. T1 of aVDAC2-FNDs from 4 patients in cGCs (C) and mGCs (D) were measured at 0, 1, 5, 10, 20, 30 minutes. The right Y axis represents the estimated radical concentration obtained from previous work [1]. Each particle is represented by one color and each curve represents measurements performed on the same particle at different time. The data were analyzed by using paired *t* test in comparison to the control groups.

1. Perona Martínez F, Nusantara AC, Chipaux M, Padamati SK, Schirhagl R: **Nanodiamond Relaxometry-Based Detection of Free-Radical Species When Produced in Chemical Reactions in Biologically Relevant Conditions**. *ACS sensors* 2020, **5**(12):3862-3869.
