# Supplementary material for: Quantum Sensing of Free Radicals in Primary Human Granulosa Cells with Nanoscale Resolution
Source: ACS Cent Sci. 2023 Aug 30;9(9):1784–98. doi: 10.1021/acscentsci.3c00747 (PMC10540281; doi:10.1021/acscentsci.3c00747)
Supplement: Supplementary file 2 — oc3c00747_si_002.pdf [file oc3c00747_si_002.pdf]

oc-2023-007475.R1

Name: Peer Review Information for "Quantum sensing of free radicals in primary human granulosa cells with nanoscale resolution"

First Round of Reviewer Comments

Reviewer: 1

Comments to the Author

The authors conducted a fascinating research approach. Combining biology/chemistry with quantum physics is highly relevant for all involved research communities. I'm strongly suggesting to publish the manuscript after a minor revision.

Q1: What is the natural T1 time of the used nanodiamonds?

Q2: The authors should add the raw T1 measurement data to the main manuscript. It is not clear from the figures (bad quality -> improvement needed before publication) how the T1 relaxation looks like. Another simple question is, how good is the fit? -> (We need values and a fitting model).

Q3: What was the laser power during the experiments of the 532 nm Laser?

Q4: What was the temperature of the nanodiamonds during the measurements?

Q5: How does the experimental setup look like? Was there an incubator involved? When yes how does it look in detail?

Reviewer: 2

Comments to the Author

The article describes a study titled "**Detection of free radicals in primary human granulosa cells using quantum sensing at the nanoscale**." The experiments conducted in the study are viable, and the reported results are plausible. The paper demonstrates good writing quality, and the findings are intriguing. No logical errors were found in the interpretation of the experimental data. Considering the content, the manuscript is a good fit for publication in ACS Central Science. Thus, I highly recommend that the manuscript should be accepted for publication, with only a minor revision required.

1. Line 39 and line 361: Using italics for "in vitro" writing is recommended.
2. Line 100-110: The fluorescent nanodiamond (FND) is unique due to its negatively charged nitrogen-vacancy (NV-) centers, which serve as built-in fluorophores, not just the nitrogen-vacancy itself. The authors can refer to the following articles for more information:

- https://doi.org/10.1021/acs.accounts.5b00484
- FND is particularly suitable for use as a contrast agent for tracking intra- and inter-cellular communications through single particle tracking. These articles provide further insights: https://doi.org/10.1002/9780470027318.a9776 https://doi.org/10.1016/j.cossms.2016.04.002
- Please include this reference (if it is relevant): the recent application of FND for COVID-19 detection https://doi.org/10.1016/j.aca.2022.340389

3. Figures 2, 4, 5, 6, and 7 should be replaced with higher-quality images. The blurriness of the words may result from the PDF conversion of the original images. Additionally, please consider increasing the font size.
4. The article needs more statistical methods. The authors should include statistical analysis methods in the Methods section and Results and Discussion. For instance, the one-way ANOVA test for statistical comparisons between different data groups.
5. Line 522: The authors should provide additional details regarding the double exponential fit. It would be helpful to explain how the constants Ca and Cb were determined and the significance of the longer constant Tb. These details would enhance readers' understanding of the authors' data interpretation.
6. The authors should address the limitations of their study. For example, they only used one type of cell line, so it remains to be seen whether the results can be generalized to other cell types. Discussing these limitations would provide readers with a broader perspective on the findings.

Author's Response to Peer Review Comments:

Dear Reviewers and Editors

First of all, I would like to thank you for your time and energy that you have invested in helping us improve our manuscript, for your constructive feedback and of course for the overwhelmingly positive response. We have addressed the comments that were raised by the reviewers in full. A point by point response is given below and the changes are marked in yellow in the main manuscript. We hope that the revised manuscript will meet your expectations and we are looking forward to your response.

Best regards Romana Schirhagl

Reviewer: 1

Recommendation: Publish in ACS Central Science after minor revisions noted.

Comments:
The authors conducted a fascinating research approach. Combining biology/chemistry with quantum physics is highly relevant for all involved research communities. I'm strongly suggesting to publish the manuscript after a minor revision.

Q1: What is the natural T1 time of the used nanodiamonds?

Assuming that natural T1 refers to T1 in cells but in absence of stimulus, the values are shown in the supplementary information (Figure S8).

Q2: The authors should add the raw T1 measurement data to the main manuscript. It is not clear from the figures (bad quality -> improvement needed before publication) how the T1 relaxation looks like.

This is shown in Figure 1C. To make this more clear we have revised the figure caption.

Another simple question is, how good is the fit? -> (We need values and a fitting model).

We have added some more information here. The process of data analysis is discussed in detail here: Vedelaar, T.A., Hamoh, T.H., Martinez, F.P., Chipaux, M. and Schirhagl, R., 2022. Optimising data processing for nanodiamond based relaxometry. arXiv preprint arXiv:2211.07269.

Q3: What was the laser power during the experiments of the 532 nm Laser?

The laser power that was used is 50 μW at the position of the sample. This information can be found in the materials and methods section.

Q4: What was the temperature of the nanodiamonds during the measurements?

All measurements were conducted at room temperature. This information has been added to the manuscript.

Q5: How does the experimental setup look like? Was there an incubator involved? When yes how does it look in detail?

There was no incubator used for this experiment (we have one but for most cells it is not needed only for very fragile cell lines or tissues). We have confirmed that the environment, thus including temperature, does not affect the T1 value for the time that we have measured (30 min) (Figure S8)

Reviewer 2

The article describes a study titled "Detection of free radicals in primary human granulosa cells using quantum sensing at the nanoscale." The experiments conducted in the study are viable, and the reported results are plausible. The paper demonstrates good writing quality, and the findings are intriguing. No logical errors were found in the interpretation of the experimental data. Considering the content, the manuscript is a good fit for publication in ACS Central Science. Thus, I highly recommend that the manuscript should be accepted for publication, with only a minor revision required.

1. Line 39 and line 361: Using italics for "in vitro" writing is recommended.

Following this suggestion, we have formatted in vitro in cursive. (here and at other places in the manuscript).

2. Line 100-110: The fluorescent nanodiamond (FND) is unique due to its negatively charged nitrogen-vacancy (NV-) centers, which serve as built-in fluorophores, not just the nitrogenvacancy itself. The authors can refer to the following articles for more information:

• https://doi.org/10.1021/acs.accounts.5b00484

This is correct. It is indeed the negatively charged NV- center that is used here. We have made the statement more precise and refer to the reference that was provided by the reviewer.

• FND is particularly suitable for use as a contrast agent for tracking intra- and inter-cellular communications through single particle tracking. These articles provide further insights:
https://doi.org/10.1002/9780470027318.a9776
https://doi.org/10.1016/j.cossms.2016.04.002

This is also correct and a very attractive and important application of nanodiamonds. We have included this information and refer to the provided articles.

• Please include this reference (if it is relevant): the recent application of FND for COVID19 detection https://doi.org/10.1016/j.aca.2022.340389

We have followed the suggestion and included the suggested paper.

3. Figures 2, 4, 5, 6, and 7 should be replaced with higher-quality images. The blurriness of the words may result from the PDF conversion of the original images. Additionally, please consider increasing the font size.

The blurriness is likely indeed due to the conversion to PDF. We have supplied high resolution images to make sure. As suggested by the reviewer, we have increased the font size to improve readability.

4. The article needs more statistical methods. The authors should include statistical analysis methods in the Methods section and Results and Discussion. For instance, the one-way ANOVA test for statistical comparisons between different data groups.

We did statistical evaluation of our results but indeed we did not make a separate section for this information. Following the suggestion of the reviewer we have added a separate section on the statistics in the materials and methods section.

5. Line 522: The authors should provide additional details regarding the double exponential fit. It would be helpful to explain how the constants Ca and Cb were determined and the significance of the longer constant Tb. These details would enhance readers' understanding of the authors' data interpretation.

This was mostly done empirically. We basically conducted measurements with different concentrated solutions and then observed how well different fit models predicted the concentrations. This came from an initial observation that the more common single exponential model fit the data poorly for ensembles. We have added some more information here. The process of data analysis is discussed in detail here: Vedelaar, T.A., Hamoh, T.H., Martinez, F.P., Chipaux, M. and Schirhagl, R., 2022. Optimising data processing for nanodiamond based relaxometry. arXiv preprint arXiv:2211.07269.

6. The authors should address the limitations of their study. For example, they only used one type of cell line, so it remains to be seen whether the results can be generalized to other cell types. Discussing these limitations would provide readers with a broader perspective on the findings.

Here is a misunderstanding: these experiments are not on a cell line but on primary cells from patients. But it is of course true, that the study is on that specific cell type. There is a section on the limitations of the study (at the end of the discussion section) but we have slightly extended it and added the argument on potential other cell types that would be interesting to look at. Most obvious here are of course the oocytes themselves which we can not investigate due to ethical reasons.

Formatting Needs:

AU EMAIL: Please include the email address of the corresponding author on the first page of the manuscript, and the Supporting Information if submitted, with an asterisk next to their name in the author list. Please be sure to label “email.”

The corresponding author emails are provided in the manuscript and the supporting information.

ABSTRACT: Please make sure the word count does not exceed 200 words.

We have shortened the abstract to meet this requirement.

SI PARAGRAPH: If the manuscript is accompanied by any supporting information for publication, a brief description of the supplementary material is required in the manuscript. The appropriate format is: Supporting Information. Brief statement in non-sentence format listing the contents of the material supplied as Supporting Information.

We have added the SI paragraph listing the content of the supplementary information.

GENERAL REF FORMATTING: Periodical references should contain authors' surnames followed by initials, article title, journal abbreviation, year, volume number, and page range. Refs with more than 10 authors should list the first 10 and then be followed by "et al."

We have made sure that references are formatted correctly.

Web sources must include access date.

We only have journal articles as references.

TOC MISSING: Provide a TOC image per journal guidelines (3.25 in. × 1.75 in. (8.25 cm × 4.45 cm) ; on the last page of the Manuscript) with the heading "TOC Graphic" above the graphic. Make sure to designate the file as "Graphic for Manuscript."

We have added A TOC graphic.

SYNOPSIS MISSING: The synopsis should be no more than 200 characters (including spaces) and should reasonably correlate with the TOC graphic. The synopsis is intended to explain the importance of the article to a broader readership across the sciences. Please place your synopsis in the manuscript file after the TOC graphic.

The missing synopsis was added.

SI PG#S: The supporting information pages must be numbered consecutively, starting with page S1.

As requested we have added page numbers to the supporting information.

oc-2023-007475.R2

Name: Peer Review Information for "Quantum sensing of free radicals in primary human granulosa cells with nanoscale resolution"

Second Round of Reviewer Comments

Reviewer: 1

Comments to the Author

The authors showed effort in improving the manuscript. Two answers from the authors are not clear and the figure quality is still poor.

Q2: The authors should add the raw T1 measurement data to the main manuscript. It is not clear from the figures (bad quality -> improvement needed before publication) how the T1 relaxation looks like.

T1 measurements are in general plotted as a probability to end up in a certain spin state. The measured counts are intresting but more intresting is a raw T1 measurement where the author shows the full decay with the probability ending up after the decay in a mixed state. Second Fig. 1c shows like most of the figures in the manuscript a bad quality. The authos should focus on improving the quality.

Q4: What was the temperature of the nanodiamonds during the measurements?

It is clear that the experiment was conducted at room temperature, but the main question is, what was the temperature of the diamond affected by laser heating ? This question is important to understand under which conditions the experiment was conducted with respect to the biological environment.

Author's Response to Peer Review Comments:

Dear Reviewers and editors,

Once again, I would like to thank you for your careful evaluation of our manuscript. We have addressed the comments that were still raised and hope that the new manuscript will meet your expectation. Below you can find the point by point response. Further, changes are marked in the manuscript in yellow.

Best regards Romana Schirhagl

Reviewer: 1

Recommendation: Publish in ACS Central Science after minor revisions noted.

Comments:
The authors showed effort in improving the manuscript. Two answers from the authors are not clear and the figure quality is still poor.

Q2: The authors should add the raw T1 measurement data to the main manuscript. It is not clear from the figures (bad quality -> improvement needed before publication) how the T1 relaxation looks like.

T1 measurements are in general plotted as a probability to end up in a certain spin state. The measured counts are intresting but more intresting is a raw T1 measurement where the author shows the full decay with the probability ending up after the decay in a mixed state. Second Fig. 1c shows like most of the figures in the manuscript a bad quality. The authos should focus on improving the quality.

We have already provided a file that is so large that it is difficult to upload. We believe that the "figure quality" is an issue of conversion into PDF. If needed we can provide larger files separately but our files already meet the resolution requirements from the journal. Further, we have added a file with raw data to the supplementary information. Further raw data is of course always available on request.

Q4: What was the temperature of the nanodiamonds during the measurements?
It is clear that the experiment was conducted at room temperature, but the main question is, what was the temperature of the diamond affected by laser heating ? This question is important to understand under which conditions the experiment was conducted with respect to the biological environment.

The temperature is not affected by laser heating (at least not to the extend that we can measure it).
We have done such a measurement for the same measurement scheme before which can be found in the supplementary information of:
Nie, L., Nusantara, A.C., Damle, V.G., Sharmin, R., Evans, E.P.P., Hemelaar, S.R., Van der Laan, K.J., Li, R., Perona Martinez, F.P., Vedelaar, T. and Chipaux, M., 2021. Quantum monitoring of cellular metabolic activities in single mitochondria. Science advances, 7(21), p.eabf0573.
We did not see any measurable increase in the temperature.

Here it needs to be said that we use a very low laser power of 50 μW (this is lower than what is typically used in physics labs for similar experiments to avoid heating).

This is measured at the position of the sample. However, this measurement is under continuous illumination. In a real T1 experiment, it is even MUCH lower than that since the majority of the experiment is dark time where the laser is off.

Another indication that we are not heating the sample to a problematic level can be seen from control experiments where we didn't do any interventions. In this case, the T1 is constant over the duration of the experiments. (if there was a problematic temperature increase, this would be visible as a decrease in T1 over the duration of the experiment)
